# Insights into the ecology, evolution, and metabolism of the widespread Woesearchaeotal lineages

**DOI:** 10.1186/s40168-018-0488-2

**Published:** 2018-06-08

**Authors:** Xiaobo Liu, Meng Li, Cindy J. Castelle, Alexander J. Probst, Zhichao Zhou, Jie Pan, Yang Liu, Jillian F. Banfield, Ji-Dong Gu

**Affiliations:** 1 Laboratory of Environmental Microbiology and Toxicology, School of Biological Sciences, Faculty of Science, The University of Hong Kong, Pokfulam Road, Hong Kong, 999077 China; 2 Institute for Advanced Study, Shenzhen University, Shenzhen, 518000 China; 3 Department of Earth and Planetary Science, University of California, Berkeley, 336 Hilgard Hall, Berkeley, CA 94720 USA; 4 Group for Aquatic Microbial Ecology (GAME), Biofilm Center, Department of Chemistry, University of Duisburg-Essen, 45141 Essen, Germany; 5 State Key Laboratory in Marine Pollution, City University of Hong Kong, Hong Kong, 999077 China

**Keywords:** Woesearchaeota, Subgroup, Ecology, Evolution, Metabolism, Methanogen

## Abstract

**Background:**

As a recently discovered member of the DPANN superphylum, Woesearchaeota account for a wide diversity of 16S rRNA gene sequences, but their ecology, evolution, and metabolism remain largely unknown.

**Results:**

Here, we assembled 133 global clone libraries/studies and 19 publicly available genomes to profile these patterns for Woesearchaeota. Phylogenetic analysis shows a high diversity with 26 proposed subgroups for this recently discovered archaeal phylum, which are widely distributed in different biotopes but primarily in inland anoxic environments. Ecological patterns analysis and ancestor state reconstruction for specific subgroups reveal that oxic status of the environments is the key factor driving the distribution and evolutionary diversity of Woesearchaeota. A selective distribution to different biotopes and an adaptive colonization from anoxic to oxic environments can be proposed and supported by evidence of the presence of ferredoxin-dependent pathways in the genomes only from anoxic biotopes but not from oxic biotopes. Metabolic reconstructions support an anaerobic heterotrophic lifestyle with conspicuous metabolic deficiencies, suggesting the requirement for metabolic complementarity with other microbes. Both lineage abundance distribution and co-occurrence network analyses across diverse biotopes confirmed metabolic complementation and revealed a potential syntrophic relationship between Woesearchaeota and methanogens, which is supported by metabolic modeling. If correct, Woesearchaeota may impact methanogenesis in inland ecosystems.

**Conclusions:**

The findings provide an ecological and evolutionary framework for Woesearchaeota at a global scale and indicate their potential ecological roles, especially in methanogenesis.

**Electronic supplementary material:**

The online version of this article (10.1186/s40168-018-0488-2) contains supplementary material, which is available to authorized users.

## Background

Archaea constitute a significant fraction of microbial biomass in the earth biosphere [1], playing crucial roles in global biogeochemical cycles [2–4]. The archaeal domain consisted originally only of cultural organisms (e.g., methanogens [5], hyperthermophiles [6], and halophiles [7]) that belong to two phyla, the Euryarchaeota and Crenarchaeota [8]. However, over the past few decades, the new addition of numerous 16S rRNA gene sequences and archaeal genomes has dramatically expanded the archaeal tree, and at least 26 new archaeal phyla have been proposed [9]. DNA sequencing techniques have greatly enriched the archaeal gene inventories [10] and uncovered important functions for Archaea in biogeochemical cycles [11], such as aerobic ammonia oxidation by Thaumarchaeota [12, 13].

Woesearchaeota (formerly named Deep-sea Hydrothermal Vent Euryarchaeota Group 6, DHVEG-6 [14]) were named to recognize the pioneering contribution of Carl Woese to archaeal phylogeny [15]. In combination with Diapherotrites, Parvarchaeota, Aenigmarchaeota, Nanohaloarchaeota, and Nanoarchaeota, they form a monophyletic super-phylum proposed as DPANN [15, 16]. Currently, Woesearchaeota are widely found in diverse environments, such as groundwater [15], surface water [17], inland soil [18], marine sediments [19, 20], freshwater sediments [21], activated sludge [22], wetland [23], hypersaline lakes [24], estuaries [25], and deep-sea hydrothermal vents [26]. Several Woesearchaeotal genomes with different completeness have also been detected from groundwater [15, 27], sediment [28, 29], and freshwater [16]. Based on metabolic predications, Castelle et al. reported that Woesearchaeota can enable carbon and hydrogen metabolism under anoxic conditions, which might associate with symbiotic and/or fermentation-based lifestyles [15]. Probst et al. [27] found that both the Calvin-Benson-Bassham cycle and the Wood-Ljungdahl pathway occurred most frequently, whereas the TCA cycle was little used in Woesearchaeota. They also noted that hydrogenase encoded in Woesearchaeota supports H_2_ as an important interspecies energy currency under high CO_2_ concentrations. Despite the abovementioned progress in Woesearchaeota, the distribution, biodiversity, and metabolism of this phylum still remain largely unknown. To date, there are more than 5000 high-quality Woesearchaeotal 16S rRNA gene sequences deposited in the SILVA SSU 128 database [30] and also some available metagenomic assembled genomes (MAGs) in the GenBank database [15]. Therefore, these datasets enable the exploration of general ecological patterns, and the updated genomes help better understanding of the potential metabolic functions of different Woesearchaeotal lineages in the global biogeochemical cycles.

Here, we retrieved current publicly available archaeal 16S rRNA gene sequences to explore the ecological patterns of Woesearchaeota within archaeal communities in different biotopes worldwide and to identify potential subgroups based on the phylogenetic and evolutionary relationships of different Woesearchaeotal lineages. A combination of bioinformatics, co-occurrence network, and metabolic analysis was used to reveal the possible syntrophic and/or mutualistic interactions of Woesearchaeota and their potential ecological roles in the biogeochemical cycles.

## Results

### Woesearchaeota are widely distributed in different biotopes with a high diversity

**Fig. 1:**
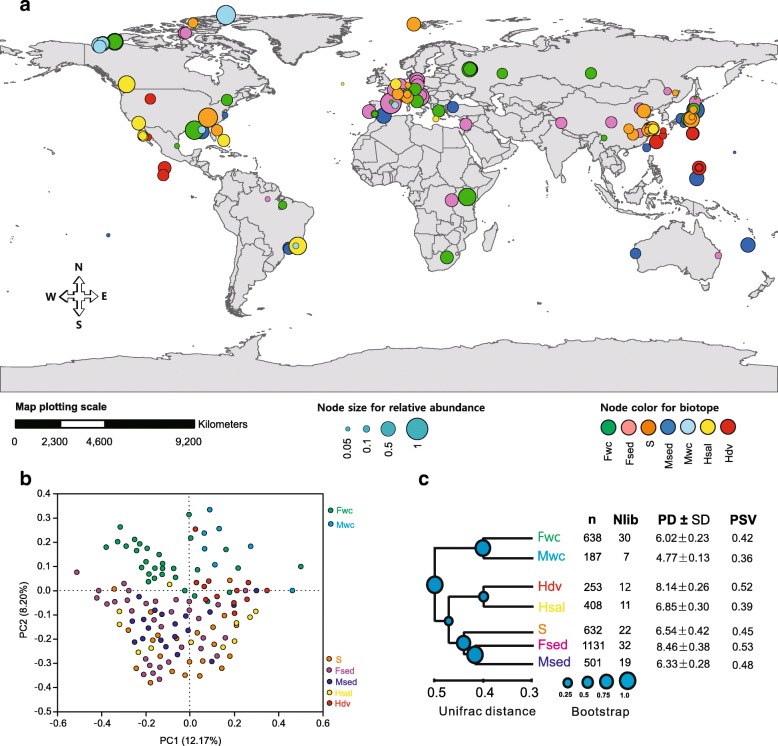
Global distribution and biodiversity patterns of Woesearchaeota in seven types of biotopes from 133 libraries/studies. **a** Global occurrence and abundance of Woesearchaeota. The abundance of 16S rRNA gene sequences of Woesearchaeota is relative to total archaea sequences in the clone libraries/studies. Each node represents one clone library/study. Node color indicates the type of biotopes, and node size represents the relative abundance in the corresponding clone libraries/studies. **b** Principal coordinate analysis (PCoA) obtained with the UniFrac distance matrix comparing the 133 libraries/studies summarized in Additional file 1: Table S1. **c** Samples clustered by the seven types of biotopes. Distances between clusters are shown in UniFrac units: a distance of 0 indicates that two environments are identical while a distance of 1 indicates that two environments contain mutually exclusive lineages. Abbreviation: n, number of sequence; Nlib, number of libraries; PD ± SD, phylogenetic diversity (PD) with standard deviation (SD); PSV, phylogenetic species variability; Fwc, freshwater; Fsed, freshwater sediment; S, soil; Msed, marine sediment; Mwc, marine water column; Hsal, hypersaline environment; and Hdv, hydrothermal vent

Woesearchaeotal 16S rRNA gene sequences from 133 public clone libraries (> 600 bp, 3584 sequences) were grouped into seven types of biotopes across the globe (Fig. 1a and Additional file 1: Table S1). Most of the samples (more than 80%) were from inland biotopes that occur in mid-latitude regions; fewer were from marine biotopes or high-latitude regions. The relative abundance of Woesearchaeota in inland biotopes (freshwater, freshwater sediment, and soil, in particular) was significantly higher than in marine biotopes (*P* < 0.05; Additional file 1: Figure S1a).

To further determine the distribution patterns of Woesearchaeota, natural samples from the 133 libraries were sorted into an ordination diagram based on the similarity of communities (Fig. 1b). Oxic biotopes were clearly separated from anoxic ones (*R*^2^ = 0.22, *P* < 0.001; Additional file 1: Figure S1b), suggesting that oxygen might be a significant factor shaping Woesearchaeotal communities.

Phylogenetic diversity (PD) indices (Fig. 1c) and rarefaction curves (Additional file 1: Figure S1c) were calculated for Woesearchaeota in each type of biotope. The rarefaction curves suggest that the Woesearchaeotal diversity is far from exhaustively sampled in anoxic biotopes, especially in freshwater sediments and extreme environments (i.e., hydrothermal vents and hypersaline biotopes). The PD value was much higher in freshwater sediment (Fsed) and hydrothermal vent (Hdv) biotopes, whereas the marine water column (Mwc) biotope held the lowest value (Fig. 1c). The mean phylogenetic species variability (PSV) value (0.45) was significantly lower than the null distribution for module 1 (0.63, *P* < 0.05) and for model 2 (0.58, *P* < 0.05; Material and Methods). Null model 1 suggests a phylogenetic clustering, indicating nonrandom relationships between phylotypes with biotopes containing more closely related phylotypes than forecast by chance [31, 32]. In contrast, null model 2 suggests a significant pattern in phylotype prevalence, where the composition of phylotypes represented nonrandom samples of the phylotype pool [31, 32]. Therefore, Fsed, Hdv, and Msed biotopes held the highest PSV values, indicating more over-dispersed phylotypes, whereas the Mwc and soil (S) biotopes had the lowest values, showing more phylogenetically clustered phylotypes. Altogether, these findings demonstrate a high biodiversity of Woesearchaeota.

### Phylogenetic analysis reveals 26 potential subgroups within the Woesearchaeota

**Fig. 2:**
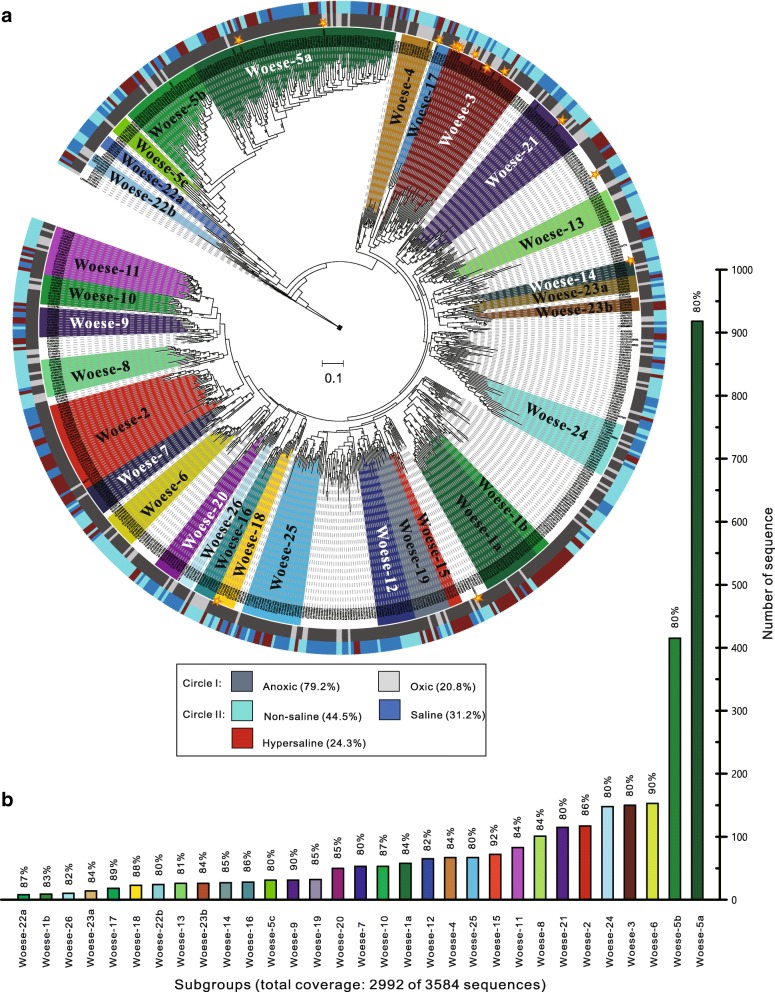
Phylogeny of 26 proposed subgroups of Woesearchaeota. **a** Maximum likelihood phylogenetic tree of Woesearchaeota based on 663 representative 16S rRNA gene sequences (> 800 bp) dereplicated at a 97% cutoff. Subgroups from Woese-1 to Woese-26 are colored within the corresponding leaves in the tree. Uncolored leaves identify sequences not assigned to any subgroup (that is, ungrouped). Outer colored circles indicate sequence origin, as follows: circle I: anoxic (dark gray), oxic (light gray); and circle II: non-saline (ice blue), saline (sky blue), hypersaline (red). Tree was drawn using the web-based interactive tree of life. The full SSU rRNA gene tree is available in result format as Additional file 2: Dataset S1. **b** Coverage and similarity of each subgroup for the total of 3584 sequences (> 600 bp) of Woesearchaeota (Additional file 4: Dataset S3). The minimum intra-group similarity (%) is listed on the top of bar for the corresponding subgroup

A total of 26 subgroups of Woesearchaeota (Woese-1 to Woese-26) were supported by both maximum likelihood (Fig. 2a and Additional file 2: Dataset S1) and Neighbor Joining distance (Additional file 1: Figure S2 and Additional file 3: Dataset S2) inferences. These subgroups covered up to 83.5% of the 3584 Woesearchaeotal 16S rRNA gene sequences, leaving 592 sequences ungrouped due to the currently incomplete topology of the phylogenetic tree (Fig. 2b and Additional file 1: Table S4). Generally, there were high intra-subgroup diversities, with the majority of subgroups holding ≤ 90% intra-group minimum similarities that ranged from 80 to 92%. The biggest sub-cluster Woese-5 split into three sister groups (Woese-5a, 5b, and 5c), each with an 80% intra-group similarity in the maximum likelihood tree (Fig. 2a). Notably, despite a stable topology of Woese-5 (bootstrap value: 92% in ML tree in Fig. 2a and 97.8% in NJ tree in Additional file 1: Figure S2), its internal branching order varied between treeing methods, so we proposed this group as a multi-furcation [33] that would be further updated after new sequences are available. Furthermore, other three subgroups (Woese-1, Woese-22, and Woese-23) split further into two sister groups.

### Oxic status drives the distribution and evolutionary diversity of organisms from different Woesearchaeotal lineages

To better summarize the range of environmental factors shaping the distribution of Woesearchaeota, we retrieved and plotted significant environmental information (that is, oxic status and salinity which are the potential forces shaping Woesearchaeotal community) around the tree (Fig. 2a). In total, there were almost 80% representative sequences originating from anoxic environments and 20% from oxic biotopes (mostly like surface freshwater and marine water). This indicates that the oxic status of an environment would be one of the major factors shaping the Woesearchaeotal communities. Moreover, the representative sequences of many subgroups (i.e., Woese-1b, 2, 9, 12, 15, 17, 18, 22a, 25) were only observed in anoxic environments, and therefore, they may be indicator species for such biotopes, but further confirmation will be required after more Woesearchaeotal sequences are added from environmental samples. Interestingly, Woese-16 was limited to sequences retrieved from oxic and non-saline biotopes (e.g., surface freshwater). For salinity, representative sequences from non-saline biotopes account for 44.5% of representative sequences, leaving 31.2 and 24.3% from saline and hypersaline environments, respectively. Two small subgroups Woese-15 and Woese-1b were only composed of sequences from hypersaline biotopes, suggesting two potential hypersaline Woesearchaeotal subgroups. Notably, Woese-2 was only observed in saline or hypersaline environments. The smallest sub-cluster Woese-22a was restricted to sequences obtained from marine sediment. All others were mixed and therefore composed of sequences from oxic, anoxic, nonsaline, saline, or hypersaline biotopes. These findings reveal that the Woesearchaeotal subgroups might be selectively distributed in local biotopes.

**Fig. 3:**
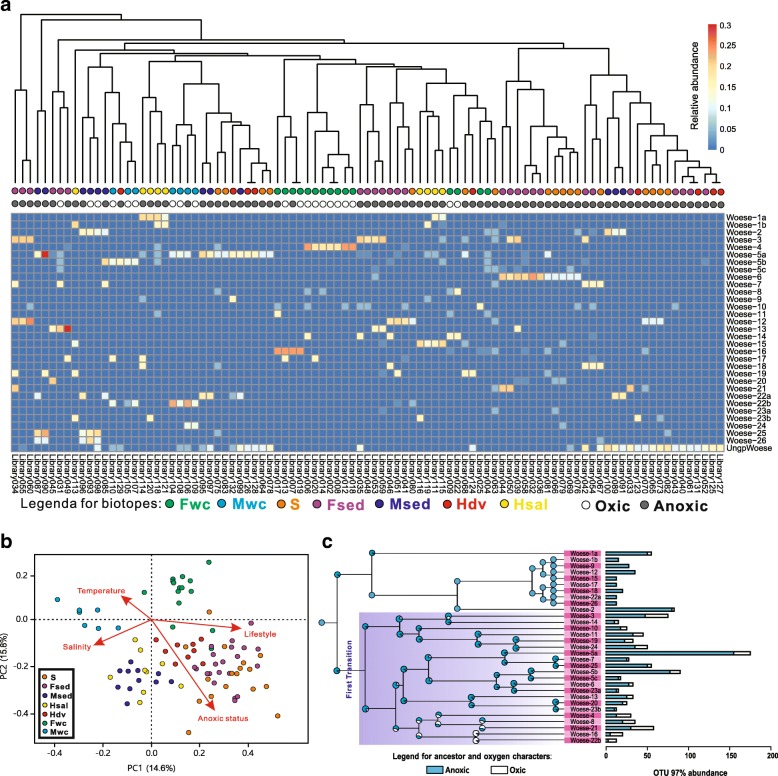
Effects of environmental factors on the distribution and evolutionary diversity of Woesearchaeotal lineages. **a** Heatmap plotting the abundance and distribution of each subgroup across 95 libraries/studies with more than 10 representative sequences of Woesearchaeota. The abundance of Woesearchaeota is relative to the total archaea sequences in the corresponding library/study. Biotope type and oxic status are expressed by colored nodes which are shown under each leaf of the cluster. **b** Principle component analysis (PCA) based on Bray-Curtis distances comparing the influences of four environmental parameters. Correlations between environmental parameters and PCA axes are represented by the length and angle of arrows. **c** Ancestral state reconstruction (ASR) of oxic status for Woesearchaeota. Pie charts on the nodes show the relative likelihoods of the two states: oxic (white) and anoxic (blue). Bar charts on the right indicate the current oxic state for Woesearchaeotal OTUs (at a 97% cutoff)

To test the above hypotheses, we investigated the relative abundance of each subgroup throughout the 95 representative libraries (Fig. 3a and Additional file 1: Table S1). Ten of 26 Woesearchaeotal subgroups showed significantly different abundance patterns in relation to oxic status (1 oxic and 9 anoxic; *P* < 0.01). Particularly, Woese-1b, Woese-3, Woese-9 Woese-12, Woese-15, Woese-18, and Woese-22a were only observed in anoxic biotopes, whereas Woese-4 and Woese-16 appeared mainly in oxic environments (Fig. 3a), such as surface freshwater and marine water. Notably, the ungrouped Woesearchaeotal lineages tend to occur in both oxic freshwater and anoxic extreme environments, such as hypersaline sites and hydrothermal vents. Other subgroups appeared in oxic or anoxic environments, but mainly in anoxic biotopes. Overall, the analysis shows that not all 26 groups and subgroups are widely distributed across the seven biotopes, and patterns related to oxic status suggest that most Woesearchaeota are anaerobes or facultative anaerobes as previously described [15].

To further confirm the above conclusion, we further compared the influences of some primary environmental factors (e.g., temperature, salinity, and oxic status) on the Woesearchaeotal assemblages by sorting them into an ordination diagram based on the phylogenetic community similarity (Fig. 3b). The principal component analysis (PCA) provided evidence for oxic status to be the first strongest factor (*R*^*2*^ = 0.642, *P* < 0.01) that explained most of the assemblages of Woesearchaeota across the subsamples (95 representative libraries), compared with other environmental factors collected. Most of Woesearchaeotal assemblages in the representative libraries were affected by both “anoxic status” and “lifestyle” (that is, living in water column, soil, or sediment; *R*^*2*^ = 0.406, *P* < 0.01), instead of “temperature” or “salinity” (*R*^*2*^ < 0.004, *P* > 0.01 for both parameters).

Given the high phylogenetic diversities and selective distribution patterns of the 26 groups and subgroups, we wondered whether evolutionary distinct Woesearchaeota lineages occur in oxic and anoxic habitats. To test this hypothesis, the ancestral state of oxygen (see the “Methods” section) was reconstructed for Woesearchaeota by using the oxic status (oxic or anoxic state) of biotope of each OTU at a species level (Fig. 3c). The ancestor state reconstruction (ASR) analysis indicated a significant relationship between differentiation patterns and oxic or anoxic biotopes where Woesearchaeota occurred. Supposing an anoxic representative as the most potential last common ancestor, this analysis supported the hypothesis of an evolutionary progression for Woesearchaeotal lineages from anoxic to oxic biotopes. After the colonization of oxic biotopes in the first transition, subsequent diversifications resulted in an increase in the number of oxic Woesearchaeotal lineages, such as Woese-16 and Woese-22b. Meanwhile, the selective distribution of Woesearchaeotal subgroups in local biotopes further supports the existence of adaptive evolution to specific environmental features, especially oxic status (oxic or anoxic state, Fig. 3a).

### Metabolic reconstruction indicates an anaerobic syntrophic lifestyle with conspicuous metabolic deficiencies

**Fig. 4:**
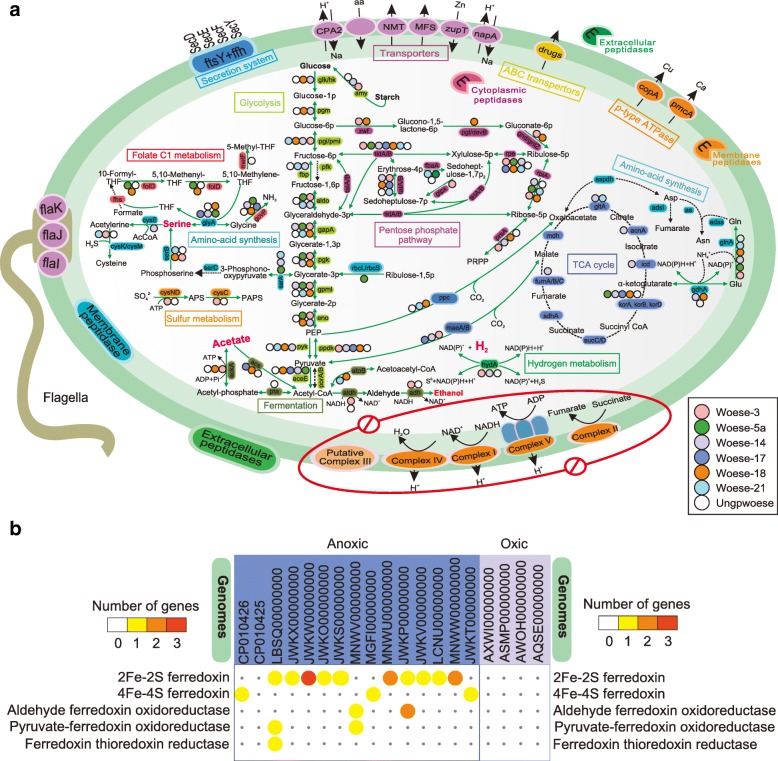
Reconstruction of metabolic pathway of Woesearchaeota in the central carbon (**a**) and comparison of genes relating to the ferredoxin-dependent pathways between organisms from oxic and anoxic biotopes (**b**). Metabolic predictions are mainly generated by referring to the interface KEGG and GenBank NCBI-nr database. Each subgroup of Woesearchaeota is depicted as a colored circle (see figure legend). Functional genes (abbreviation by referring to KEGG) encoding the relevant proteins/enzymes are labeled for each metabolic step where colored circles (that is, Woesearchaeotal subgroups) are depicted to show the potential functions of each subgroup if any. Solid arrows indicate the corresponding genes are detected for the pathways while dotted arrows indicate the corresponding genes miss from the pathways. Red “no entry” signs indicate the whole pathways missing. The crucial intermediates for methanogenesis are colored in green. All putative transporters and A-type ATPases are shown as well as secretory pathways (components of the Sec pathway) and predicted components of flagella (Additional file 1: Figure S4). Key metabolic predictions are supported by the gene information in Additional file 5: Dataset S4 and Fig. 5a. Abbreviations: THF, tetrahydrofuran; APS, adenosine 5′-phosphosulfate; PAPS, 3′-Phosphoadenosine-5′-phosphosulfate

To explore metabolic capabilities, we analyzed 19 high-quality genomes of Woesearchaeota reconstructed from previous studies (Additional file 1: Table S2). This work expands on prior metabolic predictions of Castelle et al. for nine of these genomes (e.g., AR20 in Woese-3 and AR15 in Woese-18 etc.) [15]. We assigned the 19 Woesearchaeotal members into subgroups based on their 16S rRNA gene sequences when they were available (marked in Fig. 2a and Additional file 1: Table S2). Generally, the comparative genomic analysis reveals that none of the 19 genomes appear to have the potential for a complete electron-transport chain, glycolysis, and tricarboxylic acid (TCA) cycle (Fig. 4a and Additional file 5: Dataset S4), consistent with the prior metabolic reconstructions for nine of these Woesearchaeota [15]. Identification of genes that encode flagella proteins, such as *flaI*, *flaJ*, and *flaK* (Additional file 1: Figure S4), expands the finding of potential motility and/or adhesion previously noted in Woesearchaeota (AR20, a member of Woese-3), a capacity also predicted for certain Diapherotrites [15].

**Fig. 5:**
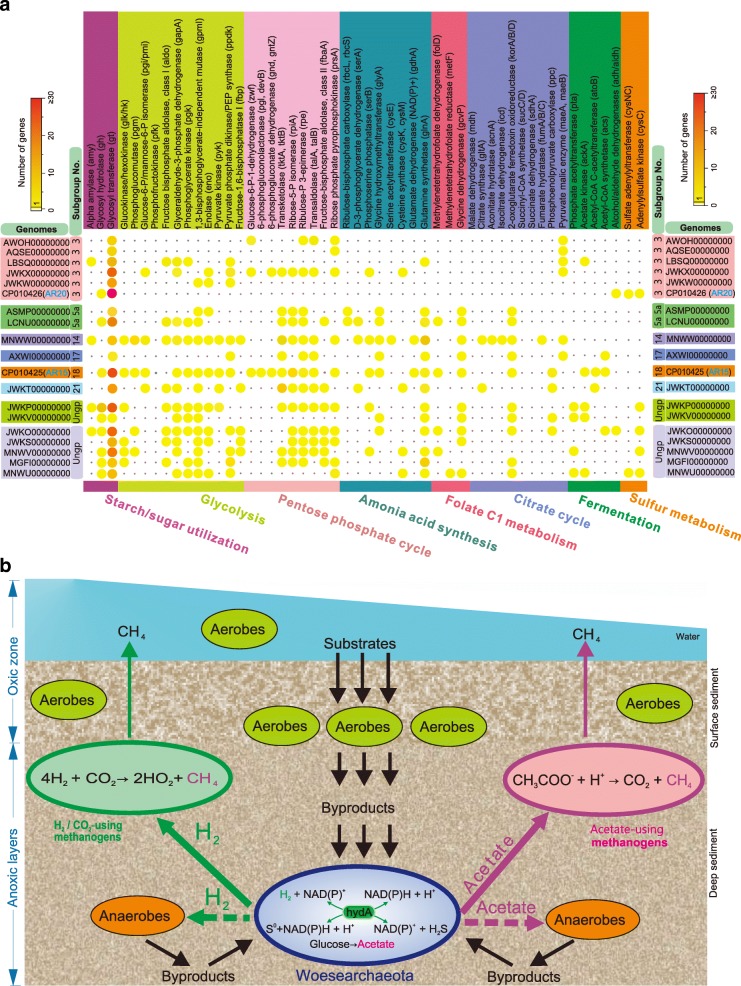
Overview of key metabolic predictions of Woesearchaeota and potential syntrophic metabolism model. **a** Comparative metabolic analyses of the 19 Woesearchaeotal genome bins generated by BLASTing against GenBank NCBI-nr database. Genes identified belong to the central carbon metabolism, nitrogen cycle, and sulfur cycle. Numbers of genes per genome matching the annotation are represented by colors in circles. **b** Syntrophic metabolic model for methanogenesis by a consortium of H_2_/CO_2_-using and acetate-using methanogens, Woesearchaeota, anaerobes, and/or aerobes. The habitat is supposed as freshwater sediments

In terms of carbon metabolism, some members of Woese-3 and Woese-14 tend to utilize starch (e.g., alpha amylase) to generate glucose for glycolysis (Figs. 4a and 5a). All Woesearchaeotal genomes lacked the gene (*pfk*) encoding phosphofructokinase to catalyze the conversion of fructose-6P to fructose-1,6P (Figs. 4a and 5a). However, they have a complete pentose phosphate pathway that enables the transformation of fructose-6P to glyceraldehyde-3P. Most can achieve the subsequent conversion from glyceraldehyde-3P to phosphoenolpyruvate (PEP), except Woese-17 and Woese-21 (Figs. 4a and 5a). Nevertheless, members in Woese-14 and Woese-18 show the potential to convert glucose to fructose-6P and subsequently complete glycolysis, with the support of a complete pentose phosphate pathway.

Notably, all 19 genomes appear to lack the gene (*porA/B*) encoding pyruvate/2-oxoacid-ferredoxin oxidoreductase, but four (members of Woese-5a, 14, 17, and 18, respectively) likely use pyruvate dehydrogenase to produce acetyl-CoA, as previously noted by Castelle et al. [15]. Members of subgroups Woese-14 and Woese-18, and Woese-18 particularly, appear to have the capacity of catalyzing some reactions of the TCA cycle, amino-acid synthesis, and folate C1 metabolism. However, after converting pyruvate to acetyl-CoA, members of these two subgroups appear not to ferment acetyl-CoA to ethanol or acetate, presumably because of the incompleteness of genomes. This process may be performed by some members of other subgroups, such as Woese-3 and the ungrouped Woesearchaeota. Generally, members of Woese-18 and Woese-21 have the gene (*atoB*) encoding acetoacetyl-CoA thiolase that converts acetyl-CoA to acetoacetyl-CoA. Apart from Woese-5a, members of other six subgroups (including the ungrouped Woesearchaeota) can provide intermediates (oxaloacetate and malate) for use in the TCA cycle or other biosynthetic pathways (e.g., amino acids conversion). For example, members in Woese-3, 14, and 17 appear to encode malate dehydrogenase to convert pyruvate to malate by fixing CO_2_ (Figs. 4a and 5a). Among them, only members in Woese-14 showed the potential to transform oxaloacetate to succinyl-CoA. Others may only metabolize α-ketoglutarate into succinyl-CoA or may participate in the branch of amino-acid metabolism from glutamic acid to glutamine.

Interestingly, genomes of three subgroups (i.e., Woese-5a, 14, and 21) encode potential D-3-phosphoglycerate dehydrogenase for the conversion of glycerate-3P into 3-phosphonooxypyruvate for amino-acid synthesis (Figs. 4a and 5a). However, this pathway appears to be blocked at the subsequent step because of the absence of phosphoserine aminotransferase in all 19 members of Woesearchaeota. Despite this, Woesearchaeota can continue the subsequent pathways to complete the biosynthesis of serine, glycine, and cysteine, and then provide a series of intermediates for methane metabolism. For example, members of three subgroups (Woese-3, 18, 21) show the potential to convert phosphoserine into serine which might be used to produce numerous intermediates for methane metabolism, such as potential 5-Methyl-THF and 5-Methyl-THMPT [34]. We did not identify the gene (*mcrA*) encoding methyl-CoM reductase, indicating that Woesearchaeota are not involved in methanogenesis. Only one member (AR20) [15] in Woese-3 and another in the ungrouped lineages might perform partial assimilatory sulfate reduction of sulfate to 3′-Phosphoadenosine-5′-phosphosulfate (PAPS) in sulfur metabolism. A few genes, such as *nir*K and *nos*Z genes, involved in nitrogen cycling are determined among the 19 genomes of Woesearchaeota, which might possibly for detoxification as proposed in previous study [15].

Consistent with the previous predictions for the first nine Woesearchaeota [15], most have anaerobic metabolisms. Interestingly, we find that members of Woesearchaeota from anoxic biotopes possess genes involved in ferredoxin-dependent pathways that are not detected in Woesearchaeota from oxic biotopes (Fig. 4b). These results further provide potential metabolic evidence for the distribution diversification of Woesearchaeotal lineages on oxic status. On the other hand, significant metabolic deficiencies and some membrane-bound hydogenases for hydrogen metabolism [15, 27] indicate that Woesearchaeota, like most other DPANN archaea [15], might perform nutritional complementation with other organisms.

### High co-occurrence with methanogenic archaea suggests the possibility of a syntrophic relationship

**Fig. 6:**
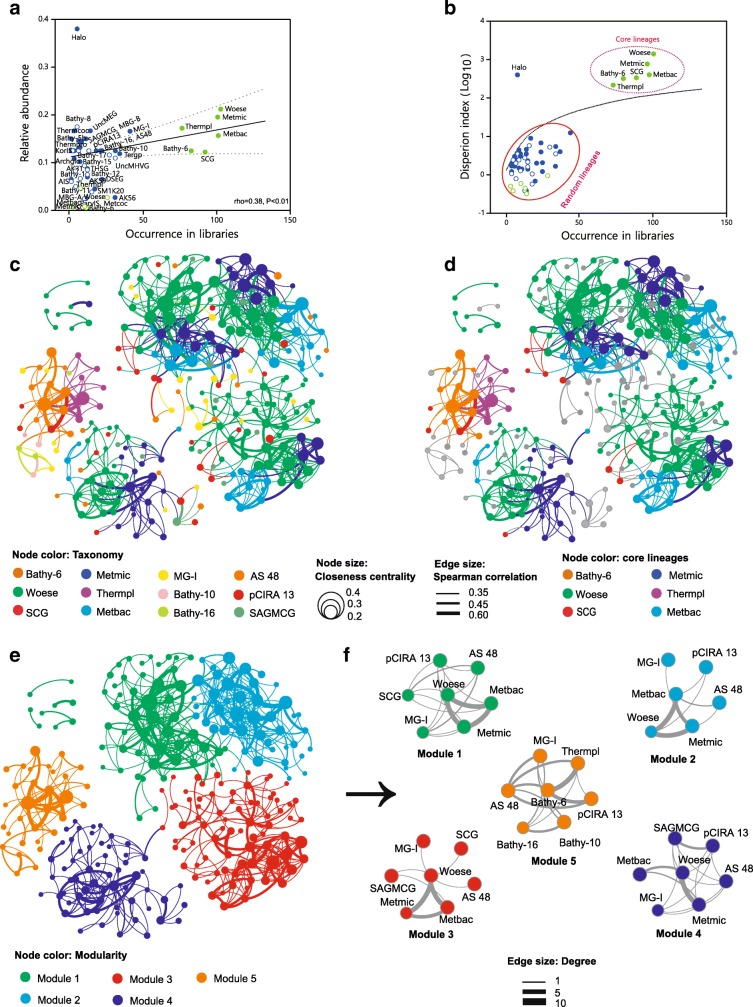
Lineage abundance distribution (LAD) and co-occurrence patterns of Woesearchaeota with other archaea across the 133 libraries/studies. **a** Occurrence of archaeal lineages (number of studies where a given lineage was found) plotted against its mean abundance across these studies. Core lineages (in green) were defined as those appearing in more than 90 libraries while satellite lineages (in blue) appearing in less than 50 libraries. Solid circles indicate lineages from anoxic biotopes while hollow circles indicate lineages from oxic biotopes. **b** Occurrence of each archaeal lineage plotted against its dispersion index. The dash line depicts the 2.5% confidence limit of the *χ*^2^ distribution suggesting that lineages falling below this line follow a Poisson distribution and are therefore randomly dispersed in space. **c** Co-occurrence network based on correlation analysis. Each node denotes an archaeal OTU at 90% cutoff. Node size indicates the closeness centrality (that is, the mean shortest path of this node to any other node), and node color represents taxonomy (see abbreviations below). Edge lines between nodes denote significant co-occurrence relationships. Edge size shows the strength of Spearman correlation among nodes. **d** Same network as **c**, but nodes are colored according to core lineages (see figure legends). **e** Same network as **c**, but nodes are colored by modules. **f** Sub-network modules clustering all OTUs belonging to the same lineage colored by modularity. Edge size indicates the number of connections (degree). Abbreviation of taxonomy: Woese, Woesearchaeota; Metmic, Methanomicrobia; Metbac, Methanobacteria; Bathy-6/10/16, Bathyarchaeota subgroup-6/10/16; Thermpl, Thermoplasmata; SCG, Soil Crenarchaeotic Group; SAGMCG, South African Gold Mine Group 1; MG-I, Marine Group I; Halobacteria, Halo; Terrestrial group, Tergp; others (Additional file 1: Table S5)

The archaeal lineage abundance distribution (LAD) showed a positive relationship between frequency of occurrence (i.e., number of libraries/studies where a specific archaeal lineage was detected) and mean relative abundance, indicating that cosmopolitan archaeal lineages were more abundant than those lineages that rarely occurred, except for Haloarchaea that appear in hypersaline ecosystems (Fig. 6a). In agreement with previous studies [35–37], archaeal lineages fall into two groups by differential occurrence: one group containing six anoxic core lineages (i.e., more frequent/abundant lineages observed in more than 90 libraries) that included Woesearchaeota that had already shown to be a core lineage in sediments [38], and another group composed of 40 rarer/less abundant satellite lineages (i.e., lineages observed in less than 50 libraries). Most frequent archaeal lineages were two anaerobic methanogenic archaeal lineages with occurrence rates of 87% (116 libraries) for Methanomicrobia and 83% (110 libraries) for Methanobacteria across the 133 libraries/studies where Woesearchaeota were detected. By mean relative abundance, Woesearchaeota were the most abundant archaeal lineage as they represented 25% of the OTUs in each library and the second most abundant were Methanomicrobia representing 20% of the OTUs in each library, where the latter were detected (Additional file 1: Table S5). The dispersion index against occurrence diagram (Fig. 6b) further supported that Woesearchaeota and other five core lineages were not randomly distributed through the libraries. Conversely, 37 of 40 satellite lineages were confirmed as randomly distributing lineages as their dispersion indices fell below the confidence of 2.5%.

After we confirmed that the LAD patterns of core archaeal lineages are certainly non-random, we further explored potential syntrophy for Woesearchaeota using co-occurrence network inference based on strong and significant Spearman correlations [39]. The network (Fig. 6c, d) was composed of 302 nodes (i.e., OTUs) and 692 edges, which presented a typical topology for a microbial network [40–42]. Within this network, Woesearchaeota were the most frequent archaeal lineages occupying 38% of the nodes and 46% of the edges, followed by methanogens (that is, Methanomicrobia and Methanobacteria with nodes: 26% and edges: 30%, in total). The network indices for the first ten highest nodes also confirmed the critical role of Woesearchaeota and methanogens in the whole network structure (Additional file 1: Table S3). Moreover, the multivariate regression tree (MRT) showed that Methanomicrobia and Methanobacteria were the most frequent core lineages to be co-indicators together with Woesearchaeota for most anoxic biotopes in paddy soil, anoxic water, and cold sediments (Additional file 1: Figure S3).

Previous studies claimed that modules could be used as ecological niches when evaluating potential functions in biological communities [43, 44]. Thus, modularity analysis was carried out, and five different modules were individually generated by assembling the OTUs that had high connections within the corresponding module (Fig. 6e) [38]. Woesearchaeota occurred in four of the five modules where they notably co-occurred more frequently with Methanomicrobia and Methanobacteria as indicated by higher degree values (that is, high level of interconnections, Fig. 6f). As suggested in the “Methods” section, each different module represented a different ecological niche [45, 46]. Therefore, the presence of Woesearchaeota-methanogens association in four different modules further supported a possible syntrophy rather than an overlap of ecological niche.

## Discussion

A survey of currently available data shows that the Woesearchaeota phylum is diverse, with at least 26 proposed subgroups distributed in different biotopes, but mainly in inland anoxic environments. Their abundance in inland over marine biotopes is in sharp contrast to current data that suggest that Bathyarchaeota and Marine Benthic Group-D are most common in marine environments [33, 47]. Our analyses suggest that the distribution of specific Woesearchaeotal subgroups associates strongly with specific biotopes, of which oxic status is the force driving the diversification and distribution of Woesearchaeota. Ferredoxin-dependent pathways consistently absent in organisms from oxic biotopes but present in organisms from anoxic biotopes metabolically support the properties of selective distribution in different environments or the corresponding adaptive strategies to oxic status of diverse biotopes (Fig. 4b).

Despite the wide distribution in seven distinct biotopes, metabolic potentials of the 19 Woesearchaeotal organisms indicate that they appear to mainly perform an anaerobic or fermentation-based lifestyle, because Woesearchaeota are found to lack a complete electron transport chain, a complete TCA cycle, and a continuous glycolysis while they do have a complete pentose phosphate pathway and some fermentation-based metabolisms. This feature would account for the ecological distribution patterns of Woesearchaeota mainly in the anoxic biotopes on Earth [15]. On the one hand, we do not deny the possibility that the absence of a complete TCA cycle or other pathways is a bias of completeness of genomes. On the other hand, we have to acknowledge the fact that the available 19 genomes cover only a small fraction of the actual diversity of Woesearcheota. But the possibility would be very little that all of the 19 genomes (including two complete genomes CP010426 and CP010425 in Table S2) commonly miss the TCA cycle or other pathways (e.g., electron transport chain or the gene porA/B encoding pyruvate/2-oxoacid-ferredoxin oxidoreductase). Conversely, all of these genomes commonly have a complete pentose phosphate pathway that enables the transformation of fructose-6P to glyceraldehyde-3P, which compensates for the absence of pathways in the glycolysis. Therefore, these metabolic deficiencies common in the 19 Woesearchaeotal organisms might indicate a potential syntrophic and/or mutualistic partnership with other organisms. Consistently, Paul et al. reported that retroelement-guided protein diversification abounding in vast lineages of uncultivated DPANN superphylum (Woesearchaeota included) may provide them with a versatile tool used for adaptation to a dynamic, host-dependent existence [48]. This nutritional dependency on other organisms would contribute to be the major challenges in the enrichment or pure cultivation of these lineages. Therefore, we suggest that the symbiosis-based approaches would benefit the success of further enrichment or pure cultivation of Woesearchaeota. This concept may also give some hints to future studies in uncultured microbes.

Co-occurrence patterns in microbial communities are typically explored to show ecological interactions between species [49, 50]. Therefore, one may wonder whether the Woesearchaeotal lineages tend to co-occur with other organisms. However, current knowledge about this is very limited. Therefore, a clear ecological relationship between Woesearchaeota and other organisms is extremely essential before assigning niches to the organisms of Woesearchaeotal lineages. For this purpose, the co-occurrence analysis has demonstrated the existence of potential consortia between Woesearchaeota and anaerobic methanogenic archaea as the core anoxic lineages in most biotopes (Fig. 6).

Given the potential of acetate fermentation and hydrogen metabolism, we therefore hypothesized the syntrophic metabolic model for a Woesearchaeota-methanogens consortium. We propose that Woesearchaeota probably support the growth of H_2_/CO_2_-using and acetate-using methanogens (Fig. 5b), enabling them to complete with hydrogenotrophic and acetotrophic methanogens [51, 52], respectively. In turn, Woesearchaeota may receive amino-acids and other compounds to compensate for their metabolic deficiencies with the help of transporters encoded in the genomes (Additional file 1: Figure S2). In addition to acetate and hydrogen, Woesearchaeota might provide methanogens with other byproducts. For example, methyl compounds in the folate C1 metabolism (Fig. 4a) might provide potential substrates for the methyl-reducing methanogens [34]. Therefore, we speculate that Woesearchaeota as partners of methanogens might directly impact the type of methanogens present in the environment and the rate of methane formation. However, further confirmation of this hypothesis could be achieved using the FISH technique or stable isotopic labeling [53, 54].

Bacteria might also play a key in the metabolic partnership with Woesearchaeota or Woesearchaeota-methanogens consortia, but this information is restricted in our study. An ideal strategy is to collectively analyze the co-occurrence of Woesearchaeota with both other archaea and bacteria across the libraries collected, which may obtain a more complete partnership involving bacteria. However, we had difficulty in acquiring the full source from the GenBank database mainly because many early studies focused only on the single communities lacking the information of either bacteria or archaea. Therefore, future complex studies will help to explore the cross-kingdom (e.g., bacteria, fungi, and protists) interactions of Woesearchaeota to better our understanding of the ecological roles of Woesearchaeota. In addition, although our study has provided ecological and genetic evidence for Woesearchaeotal metabolisms, we acknowledge that more molecular tests for specific pathways should be continued to confirm the ecological roles of Woesearchaeota in the biogeochemical cycles in the future. Therefore, all such implications will give rise to a clear orientation to exploring the ecology, evolution, and metabolic potential of Woesearchaeota in further studies.

## Methods

### Database construction

The Esearch Utility was introduced to retrieve and capture archaeal 16S rRNA gene sequences from the GenBank NCBI-nr database (by January 2017) matching the following string “16S AND 600:2000[Sequence Length] AND archaea[Organism] AND rrna[Feature key] AND isolation_source[All fields] NOT genome OR chromosome OR plasmid.” As a result, 129,622 archaeal 16S rRNA gene sequences were retrieved. Sequences that had low quality (e.g., not ribosomal or contained N in the base sequence) or lacked isolation source tags were re-checked and discarded. To retain only studies or libraries that contain Woesearchaeotal sequences, the remaining 122,559 archaeal sequences were BLASTed against SILVA SSU 128 [30, 55]. Libraries or studies with less than ten 16S rRNA gene sequences were discarded. We ended with 15,012 archaeal sequences (including 3584 Woesearchaeotal sequences longer than 600 bp) from 133 studies or libraries: 30 from freshwater (Fwc), 32 from freshwater sediment (Fsed), 22 from soil (S), 19 from marine sediment (Msed), 7 from marine water column (Mwc), 11 from hypersaline environment (Hsal), and 12 from hydrothermal vent (Hdv) (Additional file 1: Table S1). These sequences were clustered into 11,323 operational taxonomic units (OTUs) at 97% identity including 2180 OTUs belonging to Woesearchaeota (Additional file 1: Table S1).

### Phylogenetic analysis

Both neighbor-joining (see Additional file 6: Supplementary Methods) and RAxML analyses [56] were employed to construct the phylogenetic tree. Given both the topology of the tree and a good coverage of all Woesearchaeotal lineages, we determined to use 670 Woesearchaeotal representative OTU sequences that were longer than 800 bp (at 97% cutoff) to establish the phylogenetic tree. OTU representative sequences were aligned using SINA Alignment Service (https://www.arb-silva.de/aligner/) and imported into ARB software loaded [57] with the Greengenes database (Version gg_13_5, http://greengenes.secondgenome.com/downloads/database/13_5). To exclude highly variable positions, base frequency filtration was implemented by the parsimony quick add marked tool installed in ARB software before adding sequences to the maximum parsimony backbone tree. Partial sequences were discarded due to poor alignment quality or added to the tree by parsimony criteria without allowing any changes in the general topology of tree. The tree was finally constructed using 663 representative 16S rRNA gene sequences (Additional file 7: Dataset S5), including 14 sequences from the genomes (marked by stars in the tree or see Additional file 1: Table S2). Woesearchaeotal subgroup designations were made when one subgroup with > 10 representative sequences were monophyletic with both maximum likelihood and distance approaches [33]. Environmental parameters (i.e., salinity and oxygen conditions) of each sequence in the tree were collected from the “isolation sources” information on the GenBank or the corresponding publications and drawn with iTOL [58].

### Statistical analyses

In this study, the 16S rRNA gene dataset was first treated by QIIME before the downstream analyses [59]. All the sequences were treated using two approaches: phylogenetic analysis (based on the evolutionary distances computed by the RAxML tree) and taxon-based method (where taxa were captured at a given level and subsequently treated as equally divergent).

Environmental parameters of each biotope were defined mainly by referring to the corresponding literatures if any (Additional file 1: Table S1). For oxygen, if not mentioned in original references, oxic biotopes mainly refer to oxic water or surface sediments while anoxic biotopes mainly indicate anoxic groundwater, deep-sea sediments or water, soil, and hydrothermal vents. To compare the effects of these parameters on the communities of Woesearchaeota, distance matrices were constructed based on UniFrac (a beta diversity metric quantifying community similarity) for principal coordinate analysis (PCoA) of the source variations of samples [31] and Bray Curtis (a metric balancing both membership and abundance of community) for principal component analysis (PCA) of different environmental parameters [60], respectively. The permutational Manova based on 1000 permutations with the function Adonis in R package vegan was used to examine the variation source in the UniFrac matrix [61]. For PCA, Bray-Curtis dissimilarities were introduced to avoid the potential double nulls in Euclidean distances [60]. Phylogenetic diversity (PD) for each of the seven kinds of biotopes was computed as the sum of the branch length associated with the Woesearchaeotal 16S rRNA gene sequences within the corresponding biotope [32]. To reduce the bias for unequal number of sequences, the mean PD of 1000 randomized subsamples in each biotope was calculated and the SD was also shown [31]. Moreover, the phylogenetic structure for each biotope was computed using the phylogenetic species variability (PSV) index [62]. This index quantifies how phylogenetic closeness reduces the variance of a hypothetic neutral trait [31]. The value 1 indicates all species are unrelated phylogenetically while 0 shows they become more related. In order to statistically test whether biotopes harbored Woesearchaeotal species that are more or less related to each other than expected, the mean PSV with distributions of mean null values (with an iteration of 1000) was compared based on two randomization procedures: null model 1 retains species occurrence and 2 retains biotope species abundance [62]. Data were analyzed with the R package picante [63].

Taxon-based analysis was performed using QIIME based on the taxonomy of 16S rRNA reference database SILVA SSU 128 with Woesearchaeotal taxonomy updated by Additional file 8: Dataset S6. Subsequently, 11,323 archaeal OTUs (97% cutoff) belonging to 46 archaeal lineages (at a class level except Woesearchaeota at a phylum level) and from the 133 global studies/libraries were detected. A table of lineage relative abundance was reconstructed by considering the clusters subordinate to the main archaeal phyla and provided by default in the Greengenes tree [64]. This table was then used to show the lineage abundance distribution (LAD) patterns of each lineage and further determine the ecological importance of the Woesearchaeotal lineage in archaeal communities. To find out whether lineages were distributed randomly (that is, Poisson distribution), the index of dispersion for each archaeal lineage was determined as the ratio of the variance to the average relative abundance multiplied by the occurrence [38]. Lineages whose dispersion index falls between the 2.5 and 97.5% confidence interval of the *χ*^2^ distribution will follow a Poisson distribution [65].

A multivariate regression tree (MRT) was determined and generated by the R package mvpart to illustrate the relationship between the environmental matrix (Additional file 1: Table S1) and the table of lineage relative abundances [66]. Meanwhile, we introduced the indicator value (IndVal) index, which combines relative occurrence frequency and relative abundance to identify archaeal lineages, like the concept of “indicator species.”

To determine the distribution patterns of each Woesearchaeotal subgroup throughout the libraries, the OTU table of Woesearchaeota was extracted by QIIME. The OTU relative abundance table of each subgroup was reconstructed, and the relative abundance of each subgroup was calculated as the sum of the abundance of Woesearchaeotal OTU subordinate to the corresponding subgroup. To ensure a good representativeness of subject, libraries with less than ten Woesearchaeotal representative sequences were discarded here. The distribution pattern of each subgroup (including ungrouped Woesearchaeota) based on relative abundance was finally visualized on a Heatmap plot by the R statistical package ggplot2. Clustering of libraries was based on the maximum likelihood module.

An ancestral state reconstruction (ASR) was performed to test the hypothesis of a relationship between biodiversity and oxygen condition in Woesearchaeota. For each Woesearchaeotal OTU (97% cutoff), character state for oxygen was coded as 1 = oxic and 2 = anoxic. ASR was implemented using Mesquite 2.75 with the maximum likelihood module [67].

### Construction of OTU network

To identify the associations between Woesearchaeotal OTUs and other archaeal OTUs, the co-occurrence network was constructed based on the OTU occurrence frequency through the libraries. Pairwise score between OTUs represented by more than five sequences (1628 OTUs) was calculated using Spearman’s rank correlations. To ensure reliable networks, only co-occurrences satisfying a correlation with rho > 0.6 and *P* < 0.01 were considered for downstream analyses. According to random matrix theory (RMT) algorithm [45], the transition of the nearest neighbor spacing distribution (NNSD) of eigenvalues from Gaussian orthogonal ensemble (GOE) to Poisson distributions can be used as a threshold to test random co-occurrence patterns and remove random noises. A threshold of 0.90 was determined in this correlation network analysis. The network plots were visualized with the Cytoscape 2.8.3 software [46]. Nodes indicated archaeal OTUs at 97% identity while edges indicated the significant correlations between OTUs. To profile the modularity, each network was divided into individual modules. The overall topological features of different networks were described using a set of indices including degree, closeness centrality, betweenness, node degree distribution, average node connectivity, average path length, diameter, clustering coefficient, and modularity (Additional file 1: Table S3).

### Metabolic potential analysis

To explore the potential metabolic capacity of Woesearchaeota, we retrieved all their publicly available genomes from IMG/NCBI/JGI/EMBL-EBIG and finally obtained 19 genomes of Woesearchaeota for further analysis (by January 2017, Additional file 1: Table S2). Open reading frames (ORFs) were predicted on their genomic scaffolds using the metagenome mode of Prodigal and were annotated by the NCBI Prokaryotic Genome Annotation Pipeline and by the comparison with KEGG (Additional file 5: Dataset S4). Genome-based metabolic potential was determined by searching all predicted ORFs in a genome with the presence of corresponding enzymes. Metabolic processes that necessitate more than one enzyme are inferred from the presence of all corresponding enzymes (e.g., glycolysis). To infer the potential metabolic capacities of proposed subgroups of Woesearchaeota, we assigned the genomes to the corresponding groups based on their 16S rRNA gene sequences if available (marked with stars in the tree). To further identify the potential syntrophic interactions in which Woesearchaeota may be involved and, by association, in the potential metabolisms they may harbor, the key enzymes relating to metabolic processes (e.g., methanogenesis) in other high co-occurrence archaea were also explored.

## Conclusions

We investigated the ecology, evolution, and metabolism of the widespread Woesearchaeotal lineages. The ecological and phylogenetic patterns showed that members of Woesearchaeota are widely distributed in different biotopes, with a high biodiversity that reveals 26 potential subgroups, and oxic status drives their distribution and evolutionary diversity. Metabolic reconstruction for Woesearchaeota indicated an anaerobic syntrophic lifestyle with conspicuous metabolic deficiencies. Meanwhile, high co-occurrence with methanogenic archaea suggested the possibility of a syntrophic relationship between these organisms. Our findings provide an ecological and evolutionary framework for Woesearchaeota at a global scale and indicate their potential ecological roles, especially in methanogenesis.

## Additional files

Additional file 1: Supplementary Figures 1-4 and Tables 1-5. (PDF 18534 kb)

Additional file 2: Dataset S1 Maximum likelihood tree based on 663 representative 16S rRNA alignment (Additional file 7: Dataset S5), basis for Fig. 2a, in result format (663_OTU_RAxML_bipartitionsBranchLabels. result, generated by The CIPRES Science Gateway). (RESULT 39 kb)

Additional file 3: Dataset S2 Neighbor-Joining tree based on 663 representative 16S rRNA alignment (Additional file 7: Dataset S5), basis for Additional file 1: Figure S2, in newick format (663_OTU_800bp_NJ_tree.nwk, generated by MEGA7 seeing Additional file 6: Supplementary Methods). (NWK 25 kb)

Additional file 4: Dataset S3 Similarity between any 16S rRNA gene sequences of Woesearchaeota in SILVA_SSU_128 used for inferring the minimum intra-group similarity and the coverage of sequences within the corresponding subgroups in Fig. 2b and Additional file 1: Table S4. This dataset is a list. (XLSX 314 kb)

Additional file 5: Dataset S4 Functional genes annotation of 19 genomic bins by BLASTing against the GenBank NCBI-nr Dataset used for inferring the metabolic potentials in the main text Figs. 5 and 6 as well as Additional file 1: Figure S4. (XLSX 1571 kb)

Additional file 6: Supplementary Methods. (DOCX 23 kb)

Additional file 7: Dataset S5 Alignment of 663 representative 16S rRNA sequences used for inferring tree in main text Fig. 2a and Additional file 1: Figure S2. (FASTA 32892 kb)

Additional file 8: Dataset S6 Subgroup-assigned taxonomy of 663 representative 16S rRNA sequences (Woese_97_taxonomy_7_levels) used for determining the distribution patterns of each subgroup in the seven types of habitats throughout 133 libraries/studies in main text Fig. 3a and Additional file 1: Figure S3. (TXT 106 kb)
