# Supplementary material for: Insights into the ecology, evolution, and metabolism of the widespread Woesearchaeotal lineages
Source: Microbiome. 2018 Jun 8;6:102. doi: 10.1186/s40168-018-0488-2 (PMC5994134; doi:10.1186/s40168-018-0488-2)
Supplement: Supplementary file 1 — Supplementary Figures 1-4 and Tables 1-5. (PDF 18534 kb) [file 40168_2018_488_MOESM1_ESM.pdf]

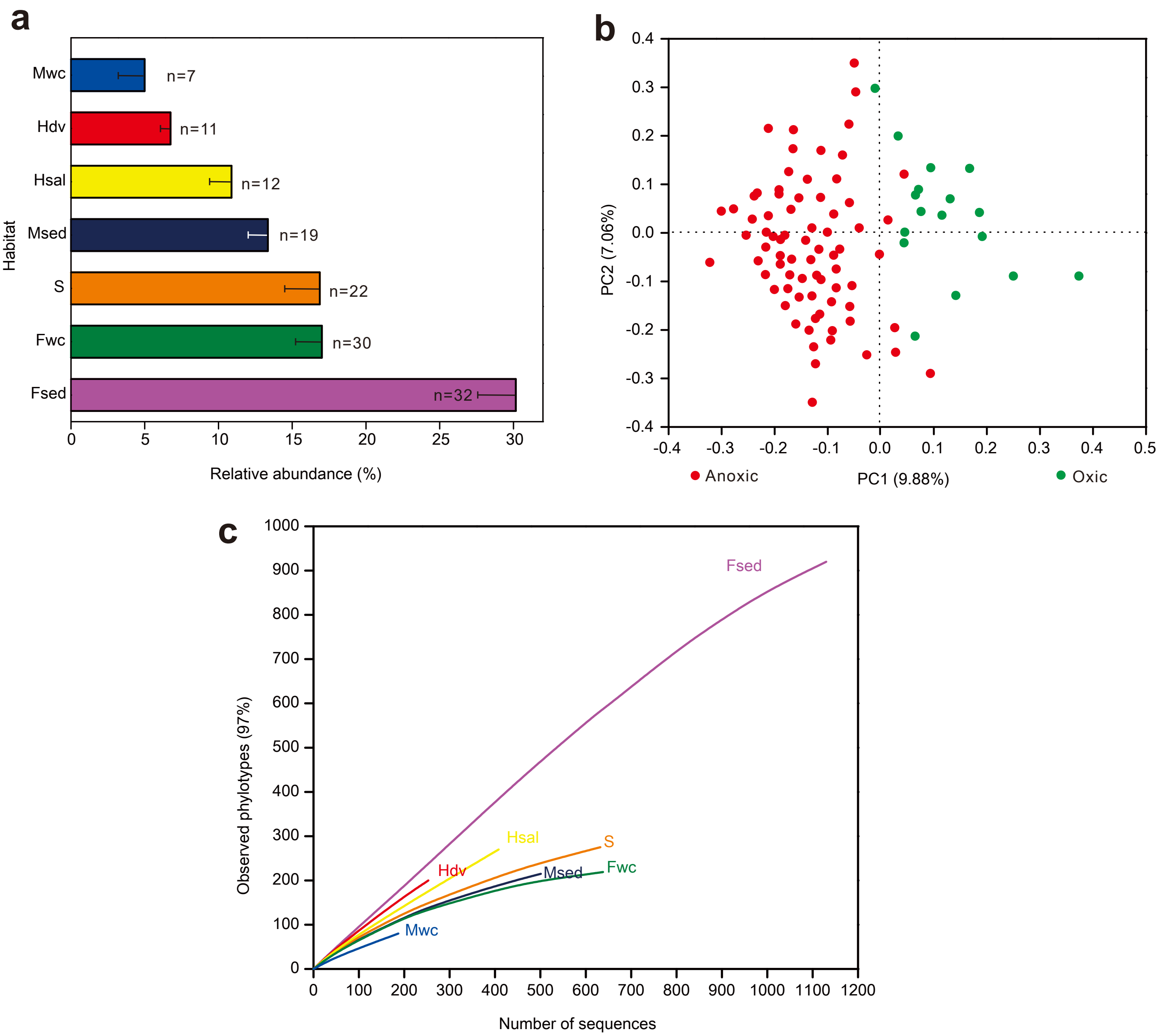

Supplementary Figure 1 | Profiles of Woesearchaeota in seven types of habitats from 133 libraries or studies. a, Relative abundance of 16S rRNA gene sequences among seven types of habitats. This abundance is based on Woesearchaeotal sequences relative to total archaea sequences within each habitat (Supplementary Table 1). **b**, Principal coordinate analysis (PCoA) plot obtained using an unweighted UniFrac distance matrix calculated on 133 clone libraries/studies associated with oxic status (anoxic: red, oxic: green). **c**, Rarefaction curves for Woesearchaeotal diversity in the seven habitats. OTUs were calculated at a 97% cutoff.

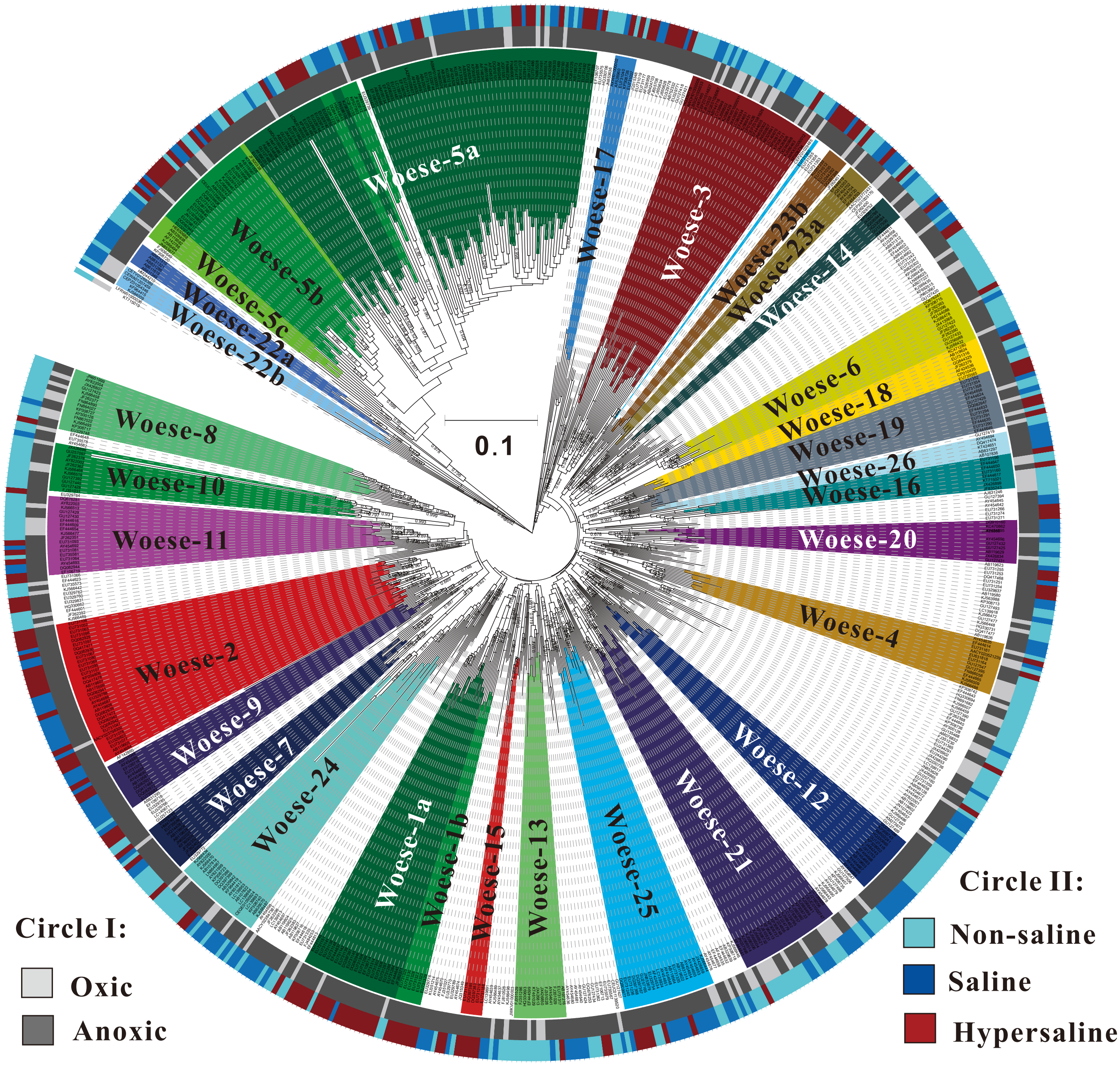

Supplementary Figure 2 | Phylogeny of 26 proposed subgroups of Woesearchaeota inferred by Neighbor-Joining method. The phylogenetic tree is based on 663 available 16S rRNA gene sequences from SILVA SSU 128 database dereplicated at a 97% cutoff. Subgroups from Woese-1 to Woese-26 are colored within the corresponding leaves in the tree. Uncolored leaves identify sequences not assigned to any subgroup (that is, ungrouped). Outer colored circles indicate sequence origin, as follows: circle I: anoxic (dark gray), oxic (light gray); and circle II: Non-saline (ice blue), saline (sky blue), hypersaline (red). Tree was drawn using the web-based interactive tree of life. The full SSU rRNA gene tree is available in newick format as Supplementary Dataset 2.

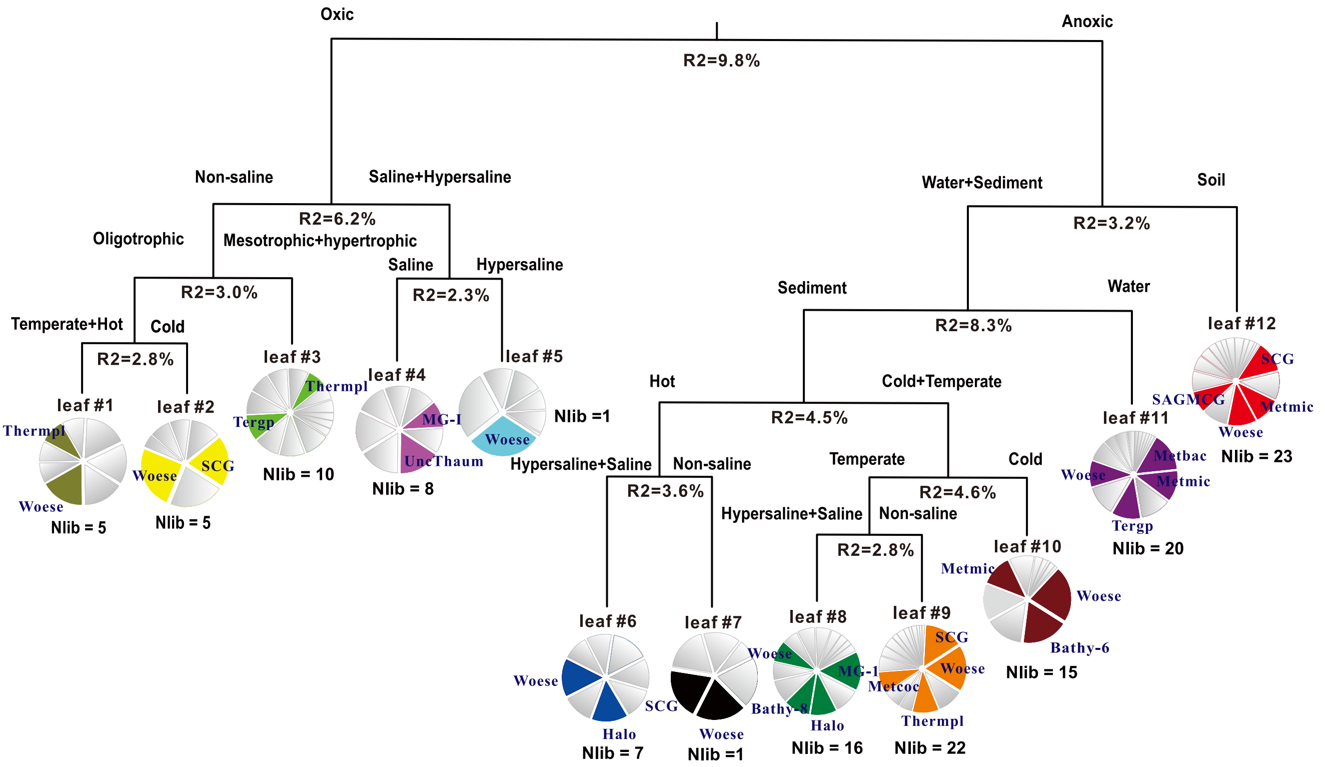

Supplementary Figure 3 | Multivariate regression tree (MRT) analysis of the interaction between archaeal lineage abundance (in term of sequence number) and environmental parameters. The model explained 51.1% of the variance in the whole data set (that is, 133 clone libraries/studies). Pies under each leaf represent the mean of normalized archaeal lineage abundance for each lineage significantly correlated with environmental parameters. Colored sections identify significant indicator lineages according to the IndVal index calculated on each leaf (P<0.01). “nlib” indicates number of clone libraries.

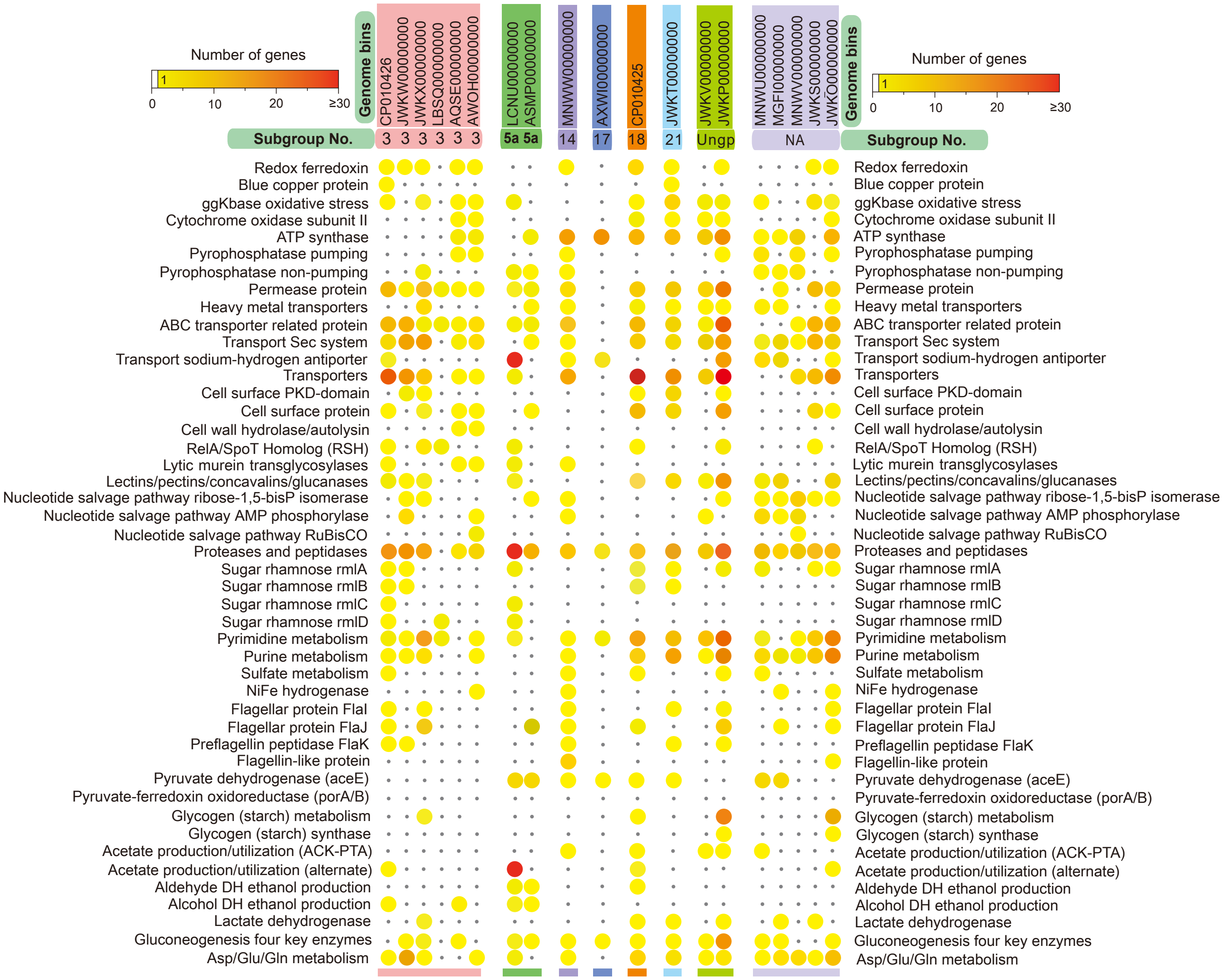

Supplementary Figure 4 | Comparative metabolic analyses of the 19 Woesearchaeotal genome bins generated by blasting against the GenBank NCBI-nr database (Relevant to Figure 5). Genes identified belong to proteins or enzymes putatively involved in electron transfer, oxidative stress, stringent response(RelA/SpoT, described in the main text), proteins involved in cell wall and cell surface biosynthesis, transporters and flagella components as well as purine and pyrimidine metabolisms. Numbers of genes per genome matching the annotation are represented by colors in circles. The lists are not mutually exclusive as a given protein can have more than one function or domain and was counted in each appropriate category. This annotation is mainly supported by Supplementary Dataset 4.

Supplementary Tables

Supplementary Table 1 Summary of the 133 archaeal 16S rRNA gene libraries included in the analysis and the environmental matrix associated

Environment	Library no.	Site location	Woese Seq.	Seq.[6]	OTU 97%	Study[7]	Temp[1]	Ls[2]	Sal[3]	Ox[4]	Ts[5]
Freshwater	1	Gold mine, South Africa	10	19	14	unpublished	1	1	1	2	1
	2	Wastewater bioreactor, China	3	32	32	unpublished	2	1	1	1	3
	3	Showerheads, USA	6	14	12	(Feazel *et al.*, 2009)	3	1	1	1	1
	4	Phreatic sinkhole (cenote), Mexico	7	78	26	(Sahl *et al.*, 2010)	1	1	1	2	1
	5	Cold freshwater spring, Italy	31	95	19	unpublished	1	1	1	1	1
	6	Lake Elementaita, Kenya	18	40	26	unpublished	2	1	1	1	2
	7	Groundwater, Japan	2	70	66	(Katsuyama *et al.*, 2013)	1	1	1	2	1
	8	Rybinsk reservoir, Russia	25	48	48	(Fedotova *et al.*, 2013)	2	1	1	1	2
	9	Ilha Grande, South Atlantic	33	460	318	(Silveira *et al.*, 2013)	1	1	1	1	1
	10	Springwater in the foot of Mt. Fuji, Japan	27	48	17	(Segawa *et al.*, 2014)	1	1	1	2	1
	11	Romashkinskoe petroleum reservoir, Russia	4	12	2	(Nazina *et al.*, 2013)	2	1	1	2	3
	12	Alpine lakes of Spanish Pyrenees, Spain	14	218	188	(Auguet and Casamayor, 2013)	2	1	1	1	1
	13	Lake Taihu, China	15	101	79	(Li *et al.*, 2015)	2	1	1	1	3
	14	Lakes Dubrovskoye and Motykino, Russia	33	62	59	(Fedotova *et al.*, 2012)	2	1	1	1	1
	15	Cold spring, China	34	47	12	unpublished	1	1	1	2	1
	16	Well 3-18-34 of Shengli Oilfield, China	4	12	7	(Mbadinga *et al.*, 2012)	3	1	1	2	3
	17	Shengli Oilfield, China	4	37	26	(Zhou *et al.*, 2012)	3	1	1	2	3
	18	Mackenzie River, Canada	22	38	20	(Galand *et al.*, 2008b)	2	1	1	1	2
	19	Mackenzie River, Canada	23	36	25	(Galand *et al.*, 2008a)	2	1	1	1	2
	20	Mackenzie River, Canada	66	89	43	(Galand *et al.*, 2006)	1	1	1	1	2

	21	Eryuan Niujie hot spring, China	3	17	2	(Sun *et al.*, 2010)	3	1	1	1	1
	22	Rio Tinto, Spain	79	620	585	(Amaral-Zettler *et al.*, 2011)	2	1	1	1	2
	23	Deep horizons of the Severnyi site, Japan	5	12	4	(Nazina *et al.*, 2010)	1	1	1	2	1
	24	Minami-Kanto Gas Field, Japan	6	29	13	(Mochimaru *et al.*, 2007)	2	1	1	2	2
	25	Tucurui hydroeletric power station reservoir, Brazil	39	182	150	(Gracas *et al.*, 2011)	2	1	1	2	2
	26	Lake Pontchartrain basin, USA	10	10	9	unpublished	2	1	1	1	2
	27	Geothermal springs, Greece	33	74	6	(Kormas *et al.*, 2009)	3	1	1	1	1
	28	Cold sulfidic springs, Germany	10	21	7	(Rudolph *et al.*, 2004)	1	1	1	1	1
	29	Alpine Lake Joeri XIII, Switzerland	4	14	13	unpublished	1	1	1	1	1
	30	Underground Crude Oil Storage Cavity, Japan	7	18	7	(Watanabe *et al.*, 2002)	1	1	1	2	3
**Freshwater sediment**	31	Pavilion Lake, Canada	22	53	53	(Chan *et al.*, 2014)	1	2	1	1	2
	32	Freshwater suboxic pond, France	14	36	30	(Bri ée *et al.*, 2007)	2	2	1	2	3
	33	Zveplenica karst spring, Slovenia	57	183	175	unpublished	1	2	1	2	1
	34	Poyang Lake, China	37	190	85	(Kou *et al.*, 2016)	2	2	1	2	2
	35	Zoige wetland in Tibetan plateau, China	90	288	269	(Fu *et al.*, 2015)	1	2	1	2	2
	36	Full-scale anaerobic digester plan, Japan	72	388	356	(Niu *et al.*, 2015)	2	2	1	2	3
	37	Red and moonmilk deposit on granite wall, Portugal	10	23	13	unpublished	1	2	1	1	1
	38	Shanghai Oil Refinery, China	4	11	7	(Wang *et al.*, 2012)	2	2	1	2	2
	39	Fen soil, Slovenia	63	82	76	unpublished	2	2	1	2	2
	40	Lakes on the Tibetan Plateau, China	21	71	40	(Liu *et al.*, 2013)	1	2	1	2	2
	41	Obukhovskoe bog, Russia	17	43	41	(Belova *et al.*, 2012)	2	2	1	2	2
	42	Char Lake on Cornwallis Island, Canada	69	227	214	(Stoeva *et al.*, 2014)	1	2	1	2	1
	43	Small Lake Wetland on Cornwallis Island, Canada	57	190	180	(Stoeva *et al.*, 2014)	1	2	1	2	1
	44	Lake Kinneret, Israel	54	161	97	(Bar-Or *et al.*, 2015)	2	2	1	2	2

45	Lake Taihu, China	35	150	111	(Fan and Wu, 2014)	2	2	1	2	3
46	Lake Kivu, East Central Africa	63	161	94	(Bhattarai *et al.*, 2012)	2	2	1	2	2
47	Wetland soil, Japan	10	36	15	(Narihiro *et al.*, 2011)	2	2	1	2	2
48	Lake Taihu, China	25	146	101	(Fan and Wu, 2014)	1	2	1	2	3
49	Lake Taihu, China	10	90	60	(Fan and Wu, 2014)	2	2	1	2	3
50	Lake Taihu, China	10	121	93	(Fan and Wu, 2014)	3	2	1	2	3
51	Beijing Cuihu Wetland Park, China	12	38	32	(Liu *et al.*, 2015)	2	2	1	2	2
52	Lake Geneva, Switzerland	11	33	27	(Haller *et al.*, 2011)	2	2	1	2	2
53	Lake Llebreta, Spain	94	97	35	(Barberán *et al.*, 2011)	2	2	1	2	2
54	Psychrophilic anaerobic reactor, China	7	49	37	(Xing *et al.*, 2010)	1	2	1	2	2
55	Slurry of biogas-reactor, China	79	946	900	unpublished	2	2	1	2	2
56	Lake Stechlin, Germany	13	25	9	(Conrad *et al.*, 2007)	2	2	1	2	1
57	Lake Dagow, Germany	12	30	19	(Chan *et al.*, 2005)	2	2	1	2	3
58	Lake Wivenhoe, Australia	9	78	41	(Green *et al.*, 2012)	2	2	1	2	3
59	Sediment microbial fuel cells, Germany	23	47	43	(De Schamphelaire *et al.*, 2010)	2	2	1	2	2
60	Drinking water reservoir, Germany	68	323	230	unpublished	2	2	1	2	2
61	Lake Batata and Lake Mussura, Germany	5	86	67	(Conrad *et al.*, 2010)	2	2	1	2	2
62	Tucurui hydroeletric power station reservoir, Brazil	8	125	11	(Gracas *et al.*, 2011)	2	2	1	2	2
63	Agricultural fields, China	48	171	93	(Ge *et al.*, 2016)	2	3	1	2	2
64	Paddy soil, China	108	456	407	(Liu *et al.*, 2016a)	2	3	1	2	2
65	Rice soil, Germany	10	115	102	(Pump *et al.*, 2015)	2	3	1	2	2
66	Qixing farmland in Sanjiang Plain, China	5	57	55	(Lu *et al.*, 2015)	2	3	1	2	2
67	Paddy soil, China	84	257	222	unpublished	2	3	1	2	2
68	Paddy soil, China	25	243	212	(Lin and Lu, 2015)	2	3	1	2	2

Soil	69	Appalachian peatland soil, USA	23	82	41	(Hawkins, 2013)	2	3	1	2	2
	70	Paddy soil, China	26	282	272	(Liu *et al.*, 2016b)	2	3	1	2	2
	71	Three Gorges Reservoir Region, China	3	13	10	unpublished	2	3	1	2	2
	72	Rice paddy field, Japan	3	21	13	(Liu *et al.*, 2012)	2	3	1	2	2
	73	Damma glacier forefield, Switzerland	17	120	93	(Zumsteg *et al.*, 2012)	1	3	1	2	2
	74	Upper Tennessee River Basin, USA	22	26	13	(Carmichael *et al.*, 2013)	1	3	1	2	1
	75	High-Arctic active permafrost soil, Canada	28	121	118	(Wilhelm *et al.*, 2011)	1	3	1	2	2
	76	Rice field soil, China	4	41	37	(Ma and Lu, 2011)	2	3	1	2	2
	77	Jidong Oilfield, China	11	26	7	(Liu *et al.*, 2009)	2	3	1	2	3
	78	Paddy soil, China	15	47	35	(Wu *et al.*, 2009)	2	3	1	2	2
	79	Upland Rice Field Soil, Japan	7	87	80	(Nishizawa *et al.*, 2008)	2	3	1	2	2
	80	Rice field soil, Italy	48	164	150	(Conrad *et al.*, 2008)	2	3	1	2	2
	81	High arctic permafrost peat, Norway	26	64	56	(Høj *et al.*, 2008)	1	3	1	2	2
	82	Rice field soil, Italy	8	23	23	(Penning and Conrad, 2006)	2	3	1	2	2
	83	Paddy soil, Japan	55	98	94	unpublished	2	3	1	2	2
	84	Paddy soil, Japan	6	18	17	unpublished	2	3	1	2	2
	85	Northeastern Gulf of Mexico, USA	105	310	90	(Moss *et al.*, 2015)	1	2	2	2	2
	86	South China Sea, China	6	41	10	unpublished	1	2	2	2	2
	87	Western Pacific, Japan	12	233	214	unpublished	1	2	2	2	2
	88	Maizuru Bay, Japan	7	26	6	unpublished	2	2	2	1	2
	89	The Pacific Ocean	14	185	179	(Wu *et al.*, 2013)	1	2	2	2	2
	90	The Pacific Ocean	38	222	206	(Wu *et al.*, 2013)	1	2	2	2	2
	91	Nankai Trough, Japan	9	74	21	(Aoki *et al.*, 2014)	1	2	2	2	2
	92	Shimizu port, Japan	3	40	9	unpublished	2	2	2	2	2

**Marine sediment**	93	The Pacific Ocean	64	132	59	(Fagervold *et al.*, 2012)	1	2	2	1	2
	94	Xiangshan Bay, China	8	47	16	(Qiu *et al.*, 2013)	2	2	2	2	2
	95	Marmara Sea, Turkey	41	141	29	(Kolukirik *et al.*, 2011)	2	2	2	2	2
	96	Seawater-processing wastewater treatment plant, Spain	63	117	106	(Sánchez *et al.*, 2011)	2	2	2	2	3
	97	Chesapeake Bay, USA	6	78	77	(Breuker *et al.*, 2011)	1	2	2	2	1
	98	Shark Bay, Western Australia	13	47	33	(Papineau *et al.*, 2005)	2	2	2	1	2
	99	Santos-Sao Vicente estuary, Brazil	31	118	87	unpublished	2	2	2	2	2
	100	Santos-Sao Vicente estuary, Brazil	35	107	66	unpublished	2	2	2	2	2
	101	The Mouth of Orikasa River, Japan	35	52	12	(Kaku *et al.*, 2005)	2	2	2	2	2
	102	West Pacific warm pool	7	14	4	(Wang *et al.*, 2005)	3	2	2	2	2
	103	Tokyo Bay, Japan	4	20	10	unpublished	2	2	2	2	2
**Marine water column**	104	Coastal Lake A of Ellesmere Island, Canada	76	89	55	(Pouliot *et al.*, 2009)	1	1	2	1	2
	105	Guanabara Bay, Brazil	14	188	130	(Turque *et al.*, 2010)	2	1	2	1	2
	106	Northeastern Gulf of Mexico, USA	15	107	41	(Moss *et al.*, 2015)	1	1	2	2	2
	107	Blanes Bay Microbial Observatory, Spain	24	234	223	(Galand *et al.*, 2010)	2	1	2	1	2
	108	Lake Mackenzie, Canada	16	40	32	(Galand *et al.*, 2008a)	1	1	2	1	2
	109	Lake Mackenzie, Canada	29	56	37	(Galand *et al.*, 2008b)	1	1	2	1	2
	110	Coastal Arctic Ocean	13	35	23	(Galand *et al.*, 2006)	1	1	2	1	2
**Hypersaline environment**	111	Guerrero Negro (GN), Mexico	227	917	807	(Robertson *et al.*, 2009)	2	2	3	2	2
	112	Cariboo Plateau, Canada	11	18	8	unpublished	1	2	3	2	2
	113	Sea port of Zeebrugge, Belgium	24	87	58	(Siegert *et al.*, 2011)	2	2	3	2	2
	114	Araruama Lagoon, Brazil	43	63	30	(Clementino *et al.*, 2008)	3	1	3	2	2
	115	Barn Island, USA	4	190	170	(Nelson *et al.*, 2009)	2	2	3	2	2

	116	Saline soil, China	11	48	32	unpublished	2	3	3	2	2
	117	Salton Sea, USA	34	77	9	(Swan *et al.*, 2010)	2	2	3	2	2
	118	Guerrero Negro, Mexico	17	99	63	(Sahl *et al.*, 2008)	2	2	3	2	2
	119	Hypersaline lake of Salt Pan, Bahamas	16	49	23	(Baumgartner *et al.*, 2009)	2	2	3	2	2
	120	Guerrero Negro, Mexico	14	61	55	(Ley *et al.*, 2006)	2	2	3	2	2
	121	Discovery Basin, Mediterranean Seafloor	2	25	25	(Hallsworth *et al.*, 2007)	2	2	3	2	2
	122	Kagoshima Bay, Western Pacific Ocean	6	14	5	(Kimura *et al.*, 2010)	3	1	2	2	2
	123	Suiyo Seamount and Myojin Knoll, Japan	33	91	26	(Takai and Horikoshi, 1999)	3	2	2	2	2
	124	Suiyo Seamount, Japan	54	160	117	(Kato *et al.*, 2012)	3	1	2	2	2
	125	Hatoma Knoll, Japan	40	97	57	(Yoshida-Takashima *et al.*, 2012)	3	2	2	2	2
	126	Inflated Plain and West Thumb regions of Yellowstone Lake, USA	52	200	192	(Kan *et al.*, 2011)	3	1	1	2	2
**Hydrothermal vent**	127	East Pacific Rise	20	51	36	(Moussard *et al.*, 2006)	3	2	2	2	2
	128	Southern Mariana Trough	10	61	36	(Kato *et al.*, 2010)	3	2	2	2	2
	129	Lau Basin	17	175	158	(Pagé *et al.*, 2008)	3	1	2	2	2
	130	Ridge Flank Abyssal Hills sea floor	8	28	15	(Ehrhardt *et al.*, 2007)	3	2	2	2	2
	131	Southern Mariana Trough	8	80	45	(Kato *et al.*, 2009)	3	2	2	2	2
	132	North and Central Big Chimney	3	40	23	(Nakagawa *et al.*, 2005a)	3	1	2	2	2
	133	North Big Chimney, Mid-Okinawa Trough	2	16	12	(Nakagawa *et al.*, 2005b)	3	1	2	2	2

Note: Environmental parameters were defined mainly by referring to the corresponding literatures if any. For oxic status, oxic habitats mainly refer to surface water or sediments whereas anoxic environments mainly refer to groundwater, deep sediments or water, soil, hydrothermal vents if the corresponding literature was unpublished.

1. Temperature: 1 = cold (<15 ^{o}C), 2 = temperate (15~30 ^{o}C), 3 = hot (>30 ^{o}C);
2. Life style: 1 = water, 2 = sediment, 3 = soil;
3. Salinity: 1 = non-saline, 2 = saline, 3 = hypersaline;
4. Oxic status: 1 = oxic, 2 = anoxic;

5. Trophic state: 1 = oligotrophic, 2 = mesotrophic, 3 = hypertrophic.
6. Libraries or studies with less than 10 archaeal 16S rRNA gene sequences were discarded from this summary.
7. Unpublished indicates direct submissions to GenBank without any published paper related.

Supplementary Table 2 Summary of the 19 genomes of Woesearchaeota

Genome ID[1]	16S rRNA gene ID[2]	Subgroup	Source	Completeness (%)	Sequencing Depth (x)	Relative GC Content (%)	Number of Protein Coding Genes	Reference
CP010426	CP010426	Woese-3	Groundwater	100	20	32	1015	(Castelle *et al.*, 2015)
JWKW00000000	JWKW01000011	Woese-3	Groundwater	78	70	34	796	(Castelle *et al.*, 2015)
JWKX00000000	JWKX01000012	Woese-3	Groundwater	87	20	30	1047	(Castelle *et al.*, 2015)
LBSQ00000000	LBSQ01000033	Woese-3	Aquifer sediment	50	9.83	37	833	(Brown *et al.*, 2015)
AQSE00000000	AQSE01000048	Woese-3	Freshwater	42	NA[3]	35	984	(Rinke *et al.*, 2013)
AWOH00000000	AWOH01000005	Woese-3	Freshwater	42	30	32	656	(Rinke *et al.*, 2013)
LCNU00000000	LCNU01000021	Woese-5a	Aquifer sediment	50	15.58	47	1033	(Brown *et al.*, 2015)
ASMP00000000	ASMP01000002	Woese-5a	Freshwater	57	NA	37	1124	(Rinke *et al.*, 2013)
MNWW00000000	KP308710	Woese-14	Groundwater	50	10	57	1475	(Probst *et al.*, 2017)
AXWI00000000	AXWI01000002	Woese-17	Freshwater	16	NA	27	462	Unpublished
CP010425	CP010425	Woese-18	Groundwater	100	15	43	1309	(Castelle *et al.*, 2015)
JWKT00000000	JWKT01000043	Woese-21	Groundwater	76	112	56	1008	(Castelle *et al.*, 2015)
JWKV00000000	JWKV01000003	UngpWoese	Groundwater	61	14	43	425	(Castelle *et al.*, 2015)
JWKP00000000	KP308716	UngpWoese	Groundwater	76	18	44.8	1243	(Castelle *et al.*, 2015)

MGFI00000000	NA	NA	Sediment	50	10	34	528	(Anantharaman *et al.*, 2016)
MNWV00000000	NA	NA	Groundwater	50	10	47	916	(Probst *et al.*, 2017)
MNWU00000000	NA	NA	Groundwater	50	10	33	1041	(Probst *et al.*, 2017)
JWKS00000000	NA	NA	Groundwater	76	12	42	577	(Castelle *et al.*, 2015)
JWKO00000000	NA	NA	Groundwater	63	11	44.6	996	(Castelle *et al.*, 2015)

Note: 1. Genome IDs referred to the accession numbers of GenBank in NCBI;

2. 16S rRNA gene IDs referred to the accession numbers in SILVA SSU_128 Dataset;

3. NA indicates the relevant information is not available.

Supplementary Table 3 Values of network indices and taxonomy for the first 10 nodes of each index

Lineage	Closeness centrality	Lineage	Betweenness centrality	Lineage	Degree
Woese	0.36	Woese	0.36	Woese	24
Woese	0.35	Woese	0.35	Woese	24
Woese	0.35	Metmic	0.33	Woese	22
Metmic	0.33	Bathy-6	0.28	Metmic	22
Woese	0.32	Metbac	0.25	Metbac	18
Metbac	0.32	Thermpl	0.25	Bathy-6	18
Metmic	0.3	Metmic	0.18	Thermpl	16
Woese	0.29	SCG	0.12	Woese	14
Bathy-6	0.28	Woese	0.08	pCIRA 13	12
Thermpl	0.27	Woese	0.08	SAGMCG	12

Abbreviations: Woese, Woesearchaeota; Metmic, Methanomicrobia; Metbac, Methanobacteria; Bathy-6, Bathyarchaeota subgroup-6; Thermpl, Thermoplasmata; SCG, Soil Crenarchaeotic Group; SAGMCG, South African Gold Mine Group 1.

Supplementary Table 4 Coverage and similarity of 26 subgroups of Woesearchaeota

Subgroup	Minimum Intra-group Similarity (%)[1]	Coverage of Sequence[2]
Woese-1a	84	58
Woese-1b	83	9
Woese-2	86	117
Woese-3	80	150
Woese-4	84	67
Woese-5a	80	918
Woese-5b	80	415
Woese-5c	80	31
Woese-6	90	153
Woese-7	80	53
Woese-8	84	101
Woese-9	90	31
Woese-10	87	53
Woese-11	84	83
Woese-12	82	65
Woese-13	81	26
Woese-14	85	27
Woese-15	92	72
Woese-16	86	28
Woese-17	89	18
Woese-18	88	23
Woese-19	85	32
Woese-20	85	50
Woese-21	80	115
Woese-22a	87	8
Woese-22b	80	24
Woese-23a	84	14
Woese-23b	84	26
Woese-24	80	148
Woese-25	80	67
Woese-26	82	10
Woese-1 to Woese-26	75	2992
Ungrouped Woese	ND[3]	592

Note: 1. Minimum intra-group similarity represents the minimum similarity between any sequences within the individual group;

2. Coverage of sequences indicates the number of 16S rRNA gene sequences (>600bp) assigned into the corresponding group with the similarity to the closest sequence in the tree above the minimum intra-group similarity;

3. ND is the abbreviation of “not determined”.

Supplementary Table 5 Occurrence of archaeal lineages across the 133 libraries/studies

Archaeal lineage (class level)	Abbrevation	Mean relative abundance	Occurrence
Aenigmarchaeota; Aenigmarchaeota Incertae Sedis	AIS	0.07	1
Bathyarchaeota; Bathy-17	Bathy-17	0.11	3
Euryarchaeota; Archaeoglobi	Archglo	0.11	3
Korarchaeota; Korarchaeota Incertae Sedis	KorIS	0.13	3
Marine Hydrothermal Vent Group(MHVG); uncultured archaeon	UncMHVG	0.13	3
Euryarchaeota; Thermococci	Thermcoc	0.17	3
Bathyarchaeota; Bathy-11	Bathy-11	0.05	4
Euryarchaeota; Methanococci	Metcoc	0.16	4
Bathyarchaeota; Bathy-8	Bathy-8	0.18	5
Euryarchaeota; Halobacteria	Halo	0.38	5
Thaumarchaeota; uncultured archaeon	UncThaum	0.13	6
Crenarchaeota; Thermoprotei	Thermpro	0.13	6
Bathyarchaeota; Bathy-5b	Bathy-5b	0.15	6
Bathyarchaeota; Bathy-15/C3	Bathy-15	0.10	7
Thaumarchaeota; South African Gold Mine Gp 1(SAGMCG-1)	SAGMCG	0.13	7
Thaumarchaeota; Marine Benthic Group B	MBG-B	0.14	8
Thaumarchaeota; Marine Benthic Group A	MBG-A	0.03	10
Parvarchaeota; Parvarchaeota Incertae Sedis	ParvIS	0.03	11
Bathyarchaeota; Bathy-14	Bathy-14	0.15	11
Bathyarchaeota; Bathy-1	Bathy-1	0.07	12
Bathyarchaeota; Bathy-12	Bathy-12	0.08	12
Thaumarchaeota; AK31	AK31	0.09	12
Aigarchaeota; Terrestrial Hot Spring Gp (THSCG)	THSG	0.09	12
Thaumarchaeota; AK56	AK56	0.03	14
Miscellaneous Euryarchaeotic Group(MEG); uncultured archaeon	UncMEG	0.17	14
Thaumarchaeota; AK59	AK59	0.06	15
SM1K20; groundwater metagenome	SM1K20	0.05	17
Bathyarchaeota; Bathy-16	Bathy-16	0.13	21
pCIRA-13; uncultured archaeon	pCIRA-13	0.13	21
Aenigmarchaeota; Deep Sea Euryarchaeotic Group(DSEG)	DSEG	0.07	22
Thaumarchaeota; AS48	AS48	0.13	22
Bathyarchaeota; Bathy-10	Bathy-10	0.13	31
Thaumarchaeota; Terrestrial group	Tergp	0.12	34
Thaumarchaeota; Marine Group I	MG-I	0.17	41
Bathyarchaeota; Bathy-6	Bathy-6	0.12	90
Euryarchaeota; Thermoplasmata	Thermpl	0.18	96
Thaumarchaeota; Soil Crenarchaeotic Group(SCG)	SCG	0.13	100

Euryarchaeota; Methanobacteria	Metbac	0.16	106
Euryarchaeota; Methanomicrobia	Metmic	0.20	112
Woesearchaeota	Woese	0.25	133

Supplementary references

Amaral-Zettler LA, Zettler ER, Theroux SM, Palacios C, Aguilera A, Amils R. (2011). Microbial community structure across the tree of life in the extreme Rio Tinto. *ISME J* **5:** 42-50.

Anantharaman K, Brown CT, Hug LA, Sharon I, Castelle CJ, Probst AJ *et al*. (2016). Thousands of microbial genomes shed light on interconnected biogeochemical processes in an aquifer system. *Nat Commun* **7**.

Aoki M, Ehara M, Saito Y, Yoshioka H, Miyazaki M, Saito Y *et al*. (2014). A long-term cultivation of an anaerobic methane-oxidizing microbial community from deep-sea methane-seep sediment using a continuous-flow bioreactor. *PLoS One* **9:** e105356.

Auguet JC, Casamayor EO. (2013). Partitioning of Thaumarchaeota populations along environmental gradients in high mountain lakes. *FEMS Microbiol Ecol* **84:** 154-164.

Bar-Or I, Ben-Dov E, Kushmaro A, Eckert W, Sivan O. (2015). Methane-related changes in prokaryotes along geochemical profiles in sediments of Lake Kinneret (Israel). *Biogeosciences* **12:** 2847-2860.

Barberán A, FERNÁNDEZ-GUERRA A, AUGUET JC, Galand PE, Casamayor EO. (2011). Phylogenetic ecology of widespread uncultured clades of the Kingdom Euryarchaeota. *Mol Ecol* **20:** 1988-1996.

Baumgartner LK, Dupraz C, Buckley DH, Spear JR, Pace NR, Visscher PT. (2009). Microbial species richness and metabolic activities in hypersaline microbial mats: insight into biosignature formation through lithification. *Astrobiology* **9:** 861-874.

Belova S, Fedotova A, Dedysh S. (2012). Prokaryotic ultramicroforms in a Sphagnum peat bog of upper Volga catchment. *Microbiology* **81:** 614-620.

Bhattarai S, Ross KA, Schmid M, Anselmetti FS, Bürgmann H. (2012). Local conditions structure unique archaeal communities in the anoxic sediments of meromictic Lake Kivu. *Microb Ecol* **64:** 291-310.

Breuker A, Köweker G, Blazejak A, Schippers A. (2011). The deep biosphere in terrestrial sediments in the Chesapeake Bay area, Virginia, USA.

Briée C, Moreira D, López-García P. (2007). Archaeal and bacterial community composition of sediment and plankton from a suboxic freshwater pond. *Res Microbiol* **158:** 213-227.

Brown CT, Hug LA, Thomas BC, Sharon I, Castelle CJ, Singh A *et al*. (2015). Unusual biology across a group comprising more than 15% of domain Bacteria. *Nature* **523:** 208-U173.

Carmichael MJ, Carmichael SK, Santelli CM, Strom A, Bräuer SL. (2013). Mn (II)-oxidizing bacteria are abundant and environmentally relevant members of ferromanganese deposits in caves of the upper Tennessee River Basin. *Geomicrobiol J* **30:** 779-800.

Castelle CJ, Wrighton KC, Thomas BC, Hug LA, Brown CT, Wilkins MJ *et al*. (2015). Genomic expansion of domain archaea highlights roles for organisms from new phyla in anaerobic carbon cycling. *Curr Biol* **25:** 690-701.

Chan OC, Claus P, Casper P, Ulrich A, Lueders T, Conrad R. (2005). Vertical distribution of structure and function of the methanogenic archaeal community in Lake Dagow sediment. *Environ Microbiol* **7:** 1139-1149.

Chan OW, Bugler-Lacap DC, Biddle JF, Lim DS, McKay CP, Pointing SB. (2014). Phylogenetic diversity of a microbialite reef in a cold alkaline freshwater lake. *Can J Microbiol* **60:** 391-398.

Clementino M, Vieira RP, Cardoso AM, Nascimento APAd, Silveira CBd, Riva TC *et al*. (2008). Prokaryotic diversity in one of the largest hypersaline coastal lagoons in the world. *Extremophiles* **12:** 595-604.

Conrad R, Chan O, Claus P, Casper P. (2007). Characterization of methanogenic Archaea and stable isotope fractionation during methane production in the profundal sediment of an oligotrophic lake (Lake Stechlin, Germany). *Limnol Oceanogr* **52:** 1393.

Conrad R, Klose M, Noll M, Kemnitz D, Bodelier PL. (2008). Soil type links microbial colonization of rice roots to methane emission. *Glob Chang Biol* **14:** 657-669.

Conrad R, Klose M, Claus P, Enrich-Prast A. (2010). Methanogenic pathway, 13C isotope fractionation, and archaeal community composition in the sediment of two clear-water lakes of Amazonia. *Limnol Oceanogr* **55:** 689-702.

De Schamphelaire L, Cabezas A, Marzorati M, Friedrich MW, Boon N, Verstraete W. (2010). Microbial community analysis of anodes from sediment microbial fuel cells powered by rhizodeposits of living rice plants. *Appl Environ Microbiol* **76:** 2002-2008.

Ehrhardt CJ, Haymon RM, Lamontagne MG, Holden PA. (2007). Evidence for hydrothermal Archaea within the basaltic flanks of the East Pacific Rise. *Environ Microbiol* **9:** 900-912.

Fagervold SK, Galand PE, Zbinden M, Gaill F, Lebaron P, Palacios C. (2012). Sunken woods on the ocean floor provide diverse specialized habitats for microorganisms. *FEMS Microbiol Ecol* **82:** 616-628.

Fan X, Wu QL. (2014). Intra-habitat differences in the composition of the methanogenic archaeal community between the Microcystis-dominated and the macrophyte-dominated bays in Taihu Lake. *Geomicrobiol J* **31:** 907-916.

Feazel LM, Baumgartner LK, Peterson KL, Frank DN, Harris JK, Pace NR. (2009). Opportunistic pathogens enriched in showerhead biofilms. *Proc Natl Acad Sci USA* **106:** 16393-16399.

Fedotova AV, Belova SE, Kulichevskaya IS, Dedysh SN. (2012). Molecular identification of filterable bacteria and archaea in the water of acidic lakes of northern Russia. *Microbiology* **81:** 281-287.

Fedotova AV, Serkebaeva YM, Sorokin VV, Dedysh SN. (2013). Filterable microbial forms in the Rybinsk water reservoir. *Microbiology* **82:** 728-734.

Fu L, Song T, Lu Y. (2015). Snapshot of methanogen sensitivity to temperature in Zoige wetland from Tibetan plateau. *Front Microbio* **6**.

Galand PE, Lovejoy C, Vincent WF. (2006). Remarkably diverse and contrasting archaeal communities in a large arctic river and the coastal Arctic Ocean. *Aquat Microb Ecol* **44:** 115-126.

Galand PE, Lovejoy C, Pouliot J, Garneau M-È, Vincent WF. (2008a). Microbial community diversity and heterotrophic production in a coastal Arctic ecosystem: A stamukhi lake and its source waters. *Limnol Oceanogr* **53:** 813-823.

Galand PE, Lovejoy C, Pouliot J, Vincent WF. (2008b). Heterogeneous archaeal communities in the particle-rich environment of an arctic shelf ecosystem. *J Mar Syst* **74:** 774-782.

Galand PE, Gutiérrez-Provecho C, Massana R, Gasol JM, Casamayor EO. (2010). Inter-annual recurrence of archaeal assemblages in the coastal NW Mediterranean Sea (Blanes Bay Microbial Observatory). *Limnol Oceanogr* **55:** 2117-2125.

Ge T, Wu X, Liu Q, Zhu Z, Yuan H, Wang W *et al*. (2016). Effect of simulated tillage on microbial autotrophic CO2 fixation in paddy and upland soils. *Sci Rep* **6**.

Gracas DA, Miranda PR, Barauna RA, McCulloch JA, Ghilardi R, Jr., Schneider MP *et al*. (2011). Microbial diversity of an anoxic zone of a hydroelectric power station reservoir in Brazilian Amazonia. *Microb Ecol* **62:** 853-861.

Green TJ, Barnes AC, Bartkow M, Gale D, Grinham A. (2012). Sediment bacteria and archaea community analysis and nutrient fluxes in a sub-tropical polymictic reservoir. *Aquat Microb Ecol* **65:** 287-302.

Høj L, Olsen RA, Torsvik VL. (2008). Effects of temperature on the diversity and community structure of known methanogenic groups and other archaea in high Arctic peat. *ISME J* **2:** 37-48.

Haller L, Tonolla M, Zopfi J, Peduzzi R, Wildi W, Pote J. (2011). Composition of bacterial and archaeal communities in freshwater sediments with different contamination levels (Lake Geneva, Switzerland). *Water Res* **45:** 1213-1228.

Hallsworth JE, Yakimov MM, Golyshin PN, Gillion JL, D'Auria G, de Lima Alves F *et al*. (2007). Limits of life in MgCl2-containing environments: chaotropicity defines the window. *Environ Microbiol* **9:** 801-813.

Hawkins AN (2013). Southern Appalachian peatlands sustain unique assemblages of Archaea, Appalachian State University.

Kaku N, Ueki A, Ueki K, Watanabe K. (2005). Methanogenesis as an important terminal electron accepting process in estuarine sediment at the mouth of Orikasa River. *Microbes Environ* **20:** 41-52.

Kan J, Clingenpeel S, Macur RE, Inskeep WP, Lovalvo D, Varley J *et al*. (2011). Archaea in yellowstone lake. *ISME J* **5:** 1784-1795.

Kato S, Kobayashi C, Kakegawa T, Yamagishi A. (2009). Microbial communities in iron-silica-rich microbial mats at deep-sea hydrothermal fields of the Southern Mariana Trough. *Environ Microbiol* **11:** 2094-2111.

Kato S, Takano Y, Kakegawa T, Oba H, Inoue K, Kobayashi C *et al*. (2010). Biogeography and biodiversity in sulfide structures of active and inactive vents at deep-sea hydrothermal fields of the Southern Mariana Trough. *Appl Environ Microbiol* **76:** 2968-2979.

Kato S, Nakawake M, Ohkuma M, Yamagishi A. (2012). Distribution and phylogenetic diversity of cbbM genes encoding RubisCO form II in a deep-sea hydrothermal field revealed by newly designed PCR primers. *Extremophiles* **16:** 277-283.

Katsuyama C, Nashimoto H, Nagaosa K, Ishibashi T, Furuta K, Kinoshita T *et al*. (2013). Occurrence and potential activity of denitrifiers and methanogens in groundwater at 140 m depth in Pliocene diatomaceous mudstone of northern Japan. *FEMS Microbiol Ecol* **86:** 532-543.

Kimura H, Mori K, Tashiro T, Kato K, Yamanaka T, Ishibashi J-I *et al*. (2010). Culture-independent estimation of optimal and maximum growth temperatures of archaea in subsurface habitats based on the G+ C content in 16S rRNA gene sequences. *Geomicrobiol J* **27:** 114-122.

Kolukirik M, Ince O, Cetecioglu Z, Celikkol S, Ince B. (2011). Spatial and temporal changes in microbial diversity of the Marmara Sea Sediments. *Mar Pollut Bull* **62:** 2384-2394.

Kormas KA, Tamaki H, Hanada S, Kamagata Y. (2009). Apparent richness and community composition of Bacteria and Archaea in geothermal springs. *Aquat Microb Ecol* **57:** 113-122.

Kou W, Zhang J, Lu X, Ma Y, Mou X, Wu L. (2016). Identification of bacterial communities in sediments of Poyang Lake, the largest freshwater lake in China. *SpringerPlus* **5:** 1.

Ley RE, Harris JK, Wilcox J, Spear JR, Miller SR, Bebout BM *et al*. (2006). Unexpected diversity and complexity of the Guerrero Negro hypersaline microbial mat. *Appl Environ Microbiol* **72:** 3685-3695.

Li J, Zhang J, Liu L, Fan Y, Li L, Yang Y *et al*. (2015). Annual periodicity in planktonic bacterial and archaeal community composition of eutrophic Lake Taihu. *Sci Rep* **5:** 15488.

Lin B, Lu Y. (2015). Bacterial and archaeal guilds associated with electrogenesis and methanogenesis in paddy field soil. *Geoderma* **259:** 362-369.

Liu GC, Tokida T, Matsunami T, Nakamura H, Okada M, Sameshima R *et al*. (2012). Microbial community composition controls the effects of climate change on methane emission from rice paddies. *Environ Microbiol Rep* **4:** 648-654.

Liu R, Zhang Y, Ding R, Li D, Gao Y, Yang M. (2009). Comparison of archaeal and bacterial community structures in heavily oil-contaminated and pristine soils. *J Biosci Bioeng* **108:** 400-407.

Liu Y, Yao T, Gleixner G, Claus P, Conrad R. (2013). Methanogenic pathways, 13C isotope fractionation, and archaeal community composition in lake sediments and wetland soils on the Tibetan Plateau. *Journal of Geophysical Research: Biogeosciences* **118:** 650-664.

Liu Y, Li H, Liu QF, Li YH. (2015). Archaeal communities associated with roots of the common reed (Phragmites australis) in Beijing Cuihu Wetland. *World J Microbiol Biotechnol* **31:** 823-832.

Liu Y, Wang P, Crowley D, Liu X, Chen J, Li L *et al*. (2016a). Methanogenic abundance and changes in community structure along a rice soil chronosequence from east China. *Eur J Soil Sci*.

Liu Y, Wang P, Pan G, Crowley D, Li L, Zheng J *et al*. (2016b). Functional and structural responses of bacterial and fungal communities from paddy fields following long-term rice cultivation. *J Soils Sed* **16:** 1460-1471.

Lu Y, Fu L, Lu Y, Hugenholtz F, Ma K. (2015). Effect of temperature on the structure and activity of a methanogenic archaeal community during rice straw decomposition. *Soil Biol Biochem* **81:** 17-27.

Ma K, Lu Y. (2011). Regulation of microbial methane production and oxidation by intermittent drainage in rice field soil. *FEMS Microbiol Ecol* **75:** 446-456.

Mbadinga SM, Li KP, Zhou L, Wang LY, Yang SZ, Liu JF *et al*. (2012). Analysis of alkane-dependent methanogenic community derived from production water of a high-temperature petroleum reservoir. *Appl Microbiol Biotechnol* **96:** 531-542.

Mochimaru H, Uchiyama H, Yoshioka H, Imachi H, Hoaki T, Tamaki H *et al*. (2007). Methanogen Diversity in Deep Subsurface Gas-Associated Water at the Minami-Kanto Gas Field in Japan. *Geomicrobiol J* **24:** 93-100.

Moss JA, McCurry C, Tominack S, Romero IC, Hollander D, Jeffrey WH *et al*. (2015). Ciliated protists from the nepheloid layer and water column of sites affected by the Deepwater Horizon oil spill in the Northeastern Gulf of Mexico. *Deep Sea Research Part I: Oceanographic Research Papers* **106:** 85-96.

Moussard H, Moreira D, Cambon-Bonavita M-A, López-García P, Jeanthon C. (2006). Uncultured Archaea in a hydrothermal microbial assemblage: phylogenetic diversity and characterization of a genome fragment from a euryarchaeote. *FEMS Microbiol Ecol* **57:** 452-469.

Nakagawa S, Takai K, Inagaki F, Chiba H, Ishibashi J-i, Kataoka S *et al*. (2005a). Variability in microbial community and venting chemistry in a sediment-hosted backarc hydrothermal system: impacts of subseafloor phase-separation. *FEMS Microbiol Ecol* **54:** 141-155.

Nakagawa S, Takai K, Inagaki F, Hirayama H, Nunoura T, Horikoshi K *et al*. (2005b). Distribution, phylogenetic diversity and physiological characteristics of epsilon-Proteobacteria in a deep-sea hydrothermal field. *Environ Microbiol* **7:** 1619-1632.

Narihiro T, Hori T, Nagata O, Hoshino T, Yumoto I, Kamagata Y. (2011). The impact of aridification and vegetation type on changes in the community structure of methane-cycling microorganisms in Japanese wetland soils. *Bioscience, biotechnology, and biochemistry* **75:** 1727-1734.

Nazina TN, Safonov AV, Kosareva IM, Ivoilov VS, Poltaraus AB, Ershov BG. (2010). Microbiological Processes in the Severnyi Deep Disposal Site for Liquid Radioactive Wastes. *Microbiology* **79:** 528-537.

Nazina TN, Shestakova NM, Pavlova NK, Tatarkin YV, Ivoilov VS, Khisametdinov MR *et al*. (2013). Functional and phylogenetic microbial diversity in formation waters of a low-temperature carbonate petroleum reservoir. *Int Biodeterior Biodegrad* **81:** 71-81.

Nelson KA, Moin NS, Bernhard AE. (2009). Archaeal diversity and the prevalence of Crenarchaeota in salt marsh sediments. *Appl Environ Microbiol* **75:** 4211-4215.

Nishizawa T, Komatsuzaki M, Kaneko N, Ohta H. (2008). Archaeal diversity of upland rice field soils assessed by the terminal restriction fragment length polymorphism method combined with real time quantitative-PCR and a clone library analysis. *Microbes Environ* **23:** 237-243.

Niu Q, Kobayashi T, Takemura Y, Kubota K, Li Y-Y. (2015). Evaluation of functional microbial community’s difference in full-scale and lab-scale anaerobic digesters feeding with different organic solid waste: Effects of substrate and operation factors. *Bioresour Technol* **193:** 110-118.

Pagé A, Tivey MK, Stakes DS, Reysenbach AL. (2008). Temporal and spatial archaeal colonization of hydrothermal vent deposits. *Environ Microbiol* **10:** 874-884.

Papineau D, Walker JJ, Mojzsis SJ, Pace NR. (2005). Composition and structure of microbial communities from stromatolites of Hamelin Pool in Shark Bay, Western Australia. *Appl Environ Microbiol* **71:** 4822-4832.

Penning H, Conrad R. (2006). Effect of inhibition of acetoclastic methanogenesis on growth of archaeal populations in an anoxic model environment. *Appl Environ Microbiol* **72:** 178-184.

Pouliot J, Galand PE, Lovejoy C, Vincent WF. (2009). Vertical structure of archaeal communities and the distribution of ammonia monooxygenase A gene variants in two meromictic High Arctic lakes. *Environ Microbiol* **11:** 687-699.

Probst AJ, Castelle CJ, Singh A, Brown CT, Anantharaman K, Sharon I *et al*. (2017). Genomic resolution of a cold subsurface aquifer community provides metabolic insights for novel microbes adapted to high CO2 concentrations. *Environ Microbiol* **19:** 459-474.

Pump J, Pratscher J, Conrad R. (2015). Colonization of rice roots with methanogenic archaea controls photosynthesis-derived methane emission. *Environ Microbiol* **17:** 2254-2260.

Qiu Q, Zhang D, Ye X, Zheng Z. (2013). The bacterial community of coastal sediments influenced by cage culture in Xiangshan Bay, Zhejiang, China. *Shengtai Xuebao/Acta Ecologica Sinica* **33:** 483-491.

Rinke C, Schwientek P, Sczyrba A, Ivanova NN, Anderson IJ, Cheng JF *et al*. (2013). Insights into the phylogeny and coding potential of microbial dark matter. *Nature* **499:** 431-437.

Robertson CE, Spear JR, Harris JK, Pace NR. (2009). Diversity and stratification of archaea in a hypersaline microbial mat. *Appl Environ Microbiol* **75:** 1801-1810.

Rudolph C, Moissl C, Henneberger R, Huber R. (2004). Ecology and microbial structures of archaeal/bacterial strings-of-pearls communities and archaeal relatives thriving in cold sulfidic springs. *FEMS Microbiol Ecol* **50:** 1-11.

Sánchez O, Garrido L, Forn I, Massana R, Maldonado MI, Mas J. (2011). Molecular characterization of activated sludge from a seawater-processing wastewater treatment plant. *Microb Biotechnol* **4:** 628-642.

Sahl JW, Pace NR, Spear JR. (2008). Comparative molecular analysis of endoevaporitic microbial communities. *Appl Environ Microbiol* **74:** 6444-6446.

Sahl JW, Fairfield N, Harris JK, Wettergreen D, Stone WC, Spear JR. (2010). Novel Microbial Diversity Retrieved by Autonomous Robotic Exploration of the World's Deepest Vertical Phreatic Sinkhole. *Astrobiology* **10** 201-213.

Segawa T, Sugiyama A, Kinoshita T, Sohrin R, Nakano T, Nagaosa K *et al*. (2014). Microbes in Groundwater of a Volcanic Mountain, Mt. Fuji; 16S rDNA Phylogenetic Analysis as a Possible Indicator for the Transport Routes of Groundwater. *Geomicrobiol J* **32:** 677-688.

Siegert M, Cichocka D, Herrmann S, Gründger F, Feisthauer S, Richnow H-H *et al*. (2011). Accelerated methanogenesis from aliphatic and aromatic hydrocarbons under iron-and sulfate-reducing conditions. *FEMS Microbiol Lett* **315:** 6-16.

Silveira CB, Cardoso AM, Coutinho FH, Lima JL, Pinto LH, Albano RM *et al*. (2013). Tropical aquatic Archaea show environment-specific community composition. *PLoS One* **8:** e76321.

Stoeva MK, Aris-Brosou S, Chételat J, Hintelmann H, Pelletier P, Poulain AJ. (2014). Microbial community structure in lake and wetland sediments from a high Arctic polar desert revealed by targeted transcriptomics. *PLoS One* **9:** e89531.

Sun P, Gu C, Ren F, Dai X, Dong Z. (2010). Prokaryotic microbial phylogenetic diversity of" Eryuan Niujie" hot spring in Yunnan province, China. *Acta Microbiol Sin* **50:** 1510-1518.

Swan BK, Ehrhardt CJ, Reifel KM, Moreno LI, Valentine DL. (2010). Archaeal and bacterial communities respond differently to environmental gradients in anoxic sediments of a California hypersaline lake, the Salton Sea. *Appl Environ Microbiol* **76:** 757-768.

Takai K, Horikoshi K. (1999). Genetic diversity of archaea in deep-sea hydrothermal vent environments. *Genetics* **152:** 1285-1297.

Turque AS, Batista D, Silveira CB, Cardoso AM, Vieira RP, Moraes FC *et al*. (2010). Environmental shaping of sponge associated archaeal communities. *PLoS One* **5:** e15774.

Wang L-Y, Li W, Mbadinga SM, Liu J-F, Gu J-D, Mu B-Z. (2012). Methanogenic microbial community composition of oily sludge and its enrichment amended with alkanes incubated for over 500 days. *Geomicrobiol J* **29:** 716-726.

Wang P, Xiao X, Wang F. (2005). Phylogenetic analysis of Archaea in the deep-sea sediments of west Pacific Warm Pool. *Extremophiles* **9:** 209-217.

Watanabe K, Kodama Y, Hamamura N, Kaku N. (2002). Diversity, Abundance, and Activity of Archaeal Populations in Oil-Contaminated Groundwater Accumulated at the Bottom of an Underground Crude Oil Storage Cavity. *Appl Environ Microbiol* **68:** 3899-3907.

Wilhelm RC, Niederberger TD, Greer C, Whyte LG. (2011). Microbial diversity of active layer and permafrost in an acidic wetland from the Canadian High Arctic. *Can J Microbiol* **57:** 303-315.

Wu L, Ma K, Li Q, Ke X, Lu Y. (2009). Composition of archaeal community in a paddy field as affected by rice cultivar and N fertilizer. *Microb Ecol* **58:** 819-826.

Wu Y-H, Liao L, Wang C-S, Ma W-L, Meng F-X, Wu M *et al*. (2013). A comparison of microbial communities in deep-sea polymetallic nodules and the surrounding sediments in the Pacific Ocean. *Deep Sea Research Part I: Oceanographic Research Papers* **79:** 40-49.

Xing W, Zhao Y, Zuo J-E. (2010). Microbial activity and community structure in a lake sediment used for psychrophilic anaerobic wastewater treatment. *J Appl Microbiol* **109:** 1829-1837.

Yoshida-Takashima Y, Nunoura T, Kazama H, Noguchi T, Inoue K, Akashi H *et al*. (2012). Spatial distribution of viruses associated with planktonic and attached microbial communities in hydrothermal environments. *Appl Environ Microbiol* **78:** 1311-1320.

Zhou L, Li KP, Mbadinga SM, Yang SZ, Gu JD, Mu BZ. (2012). Analyses of n-alkanes degrading community dynamics of a high-temperature methanogenic consortium enriched from production water of a petroleum reservoir by a combination of molecular techniques. *Ecotoxicology* **21:** 1680-1691.

Zumsteg A, Luster J, Göransson H, Smittenberg RH, Brunner I, Bernasconi SM *et al*. (2012). Bacterial, archaeal and fungal succession in the forefield of a receding glacier. *Microb Ecol* **63:** 552-564.
