# Supplementary material for: Insights into the ecology, evolution, and metabolism of the widespread Woesearchaeotal lineages
Source: Microbiome. 2018 Jun 8;6:102. doi: 10.1186/s40168-018-0488-2 (PMC5994134; doi:10.1186/s40168-018-0488-2)
Supplement: Supplementary file 6 — Supplementary Methods. (DOCX 23 kb) [file 40168_2018_488_MOESM6_ESM.docx]

**Insights into the ecology, evolution and metabolism of the widespread Woesearchaeotal lineages**

**Xiaobo Liu, Meng Li*, Cindy J. Castelle, Alexander J. Probst, Zhichao Zhou, Jie Pan, Yang Liu, Jillian F. Banfield & Ji-Dong Gu***

*For correspondence: jdgu@hku.hk (J-D Gu) or [limeng848@szu.edu.cn](mailto:limeng848@szu.edu.cn) (M Li)

**Supplementary Methods**

**Neighbor-Joining-based phylogenetic analysis for Woesearchaeota.** The evolutionary history was inferred using the Neighbor-Joining method ([Saitou and Nei, 1987](#_ENREF_5)). The optimal tree with the sum of branch length = 41.63100392 is shown. The percentage of replicate trees in which the associated taxa clustered together in the bootstrap test (1000 replicates) are shown next to the branches ([Felsenstein, 1985](#_ENREF_2)). The tree is drawn to scale, with branch lengths in the same units as those of the evolutionary distances used to infer the phylogenetic tree. The evolutionary distances were computed using the Jukes-Cantor method ([Jukes and Cantor, 1969](#_ENREF_3)) and are in the units of the number of base substitutions per site. The rate variation among sites was modeled with a gamma distribution (shape parameter = 1). The analysis involved 663 nucleotide sequences. All ambiguous positions were removed for each sequence pair. There were a total of 1393 positions in the final dataset. Evolutionary analyses were conducted in MEGA7 ([Kumar *et al.*, 2016](#_ENREF_4)). Disclaimer: Although utmost care has been taken to ensure the correctness of the caption, the caption text is provided "as is" without any warranty of any kind. Authors advise the user to carefully check the caption prior to its use for any purpose and report any errors or problems to the authors immediately (www.megasoftware.net). In no event shall the authors and their employers be liable for any damages, including but not limited to special, consequential, or other damages. Authors specifically disclaim all other warranties expressed or implied, including but not limited to the determination of suitability of this caption text for a specific purpose, use, or application.

**RAxML (Randomized Axelerated Maximum Likelihood) phylogenetic analysis for Woesearchaeota.** The RAxML tree of Woesearchaeota was generated by using the tool of RAxML-HPC BlackBox via the online system (the CIPRES Science Gateway). Rapid bootstrap heuristics were used for RAxML, which are more than an order of magnitude faster than current algorithms. Computational experiments on 22 DNA and AA (amino acid) containing 125 up to 7764 sequences; the RBS inferences are between 8 and 20 times faster (average 14.73) than SBS analyses with RAxML and between 18 and 495 times faster than BS analyses with competing programs, such as PHYML or GARLI The performance improvement increases with alignment size. For details, please refer to the 16S rRNA gene sequence-based method by [Stamatakis](javascript:;), 2014 ([Stamatakis, 2014](#_ENREF_6)).

**Multivariate regression analysis of Woesearchaeota.** According to the widespread distribution patterns, it seems that Woesearchaeota more tends to co-occur with Methanomicrobia and/or Methanobacteria in anoxic environments as these methanogetic archaeal lineages held a closer occurrence rate with Woesearchaeota compared with other archaeal lineages. To test this hypothesis and link the abundance of the archaeal lineages to environmental factors, a multivariate regression tree (MRT) was constructed (Additional file 2: Figure S3). The MRT analysis generated a twelve-leaf tree ordination based on oxic status for the first two nodes, totally explaining 51.1% of the phylogenetic variance. Therefore, anoxia tended to combine Hdv and Msed (hot- and cold-temperate anoxic marine sites), S (soil with a depth of more than 10 cm) and anoxic water-sediment, as well as Fsed and Fwc (sediments and water column from anoxic freshwaters), which agrees with previous study ([Auguet *et al.*, 2010](#_ENREF_1)). Libraries clustered in the tree leaves according to their sources (Additional file 2: Table S1). Pie charts under each leaf indicate how the relative abundance of archaeal lineages contributed to the structure of the leaves. The IndVal index for each leaf (data not shown) revealed that most core lineages are still indicator lineages (*P*<0.01), which are labeled on each pie. The Woesearchaeota was the only lineage to be indicator for all anoxic leaves and also the co-indicator lineage with Methanomicrobia in paddy soil, anoxic water and cold sediments.

**Supplementary references**

Auguet JC, Barberan A, Casamayor EO. (2010). Global ecological patterns in uncultured Archaea. *ISME J* **4:** 182-190.

Felsenstein J. (1985). Confidence limits on phylogenies: an approach using the bootstrap. *Evolution* **39:** 783-791.

Jukes TH, Cantor CR. (1969). Evolution of protein molecules. *Mammalian protein metabolism* **3:** 132.

Kumar S, Stecher G, Tamura K. (2016). MEGA7: Molecular Evolutionary Genetics Analysis version 7.0 for bigger datasets. *Mol Biol Evol* **33:** 1870-1874.

Saitou N, Nei M. (1987). The neighbor-joining method: a new method for reconstructing phylogenetic trees. *Mol Biol Evol* **4:** 406-425.

Stamatakis A. (2014). RAxML version 8: a tool for phylogenetic analysis and post-analysis of large phylogenies. *Bioinformatics* **30:** 1312-1313.
